# Supplementary material for: Antigen and checkpoint receptor engagement recalibrates T cell receptor signal strength
Source: Immunity. 2021 Nov 9;54(11):2481–2496.e6. doi: 10.1016/j.immuni.2021.08.020 (PMC8585507; doi:10.1016/j.immuni.2021.08.020)
Supplement: Document S1. Figures S1–S7 [file mmc1.pdf]

Immunity, Volume *54*

Supplemental information

Antigen and checkpoint receptor engagement recalibrates T cell receptor signal strength

Thomas A.E. Elliot, Emma K. Jennings, David A.J. Lecky, Natasha Thawait, Adriana Flores-Langarica, Alastair Copland, Kendle M. Maslowski, David C. Wraith, and David Bending

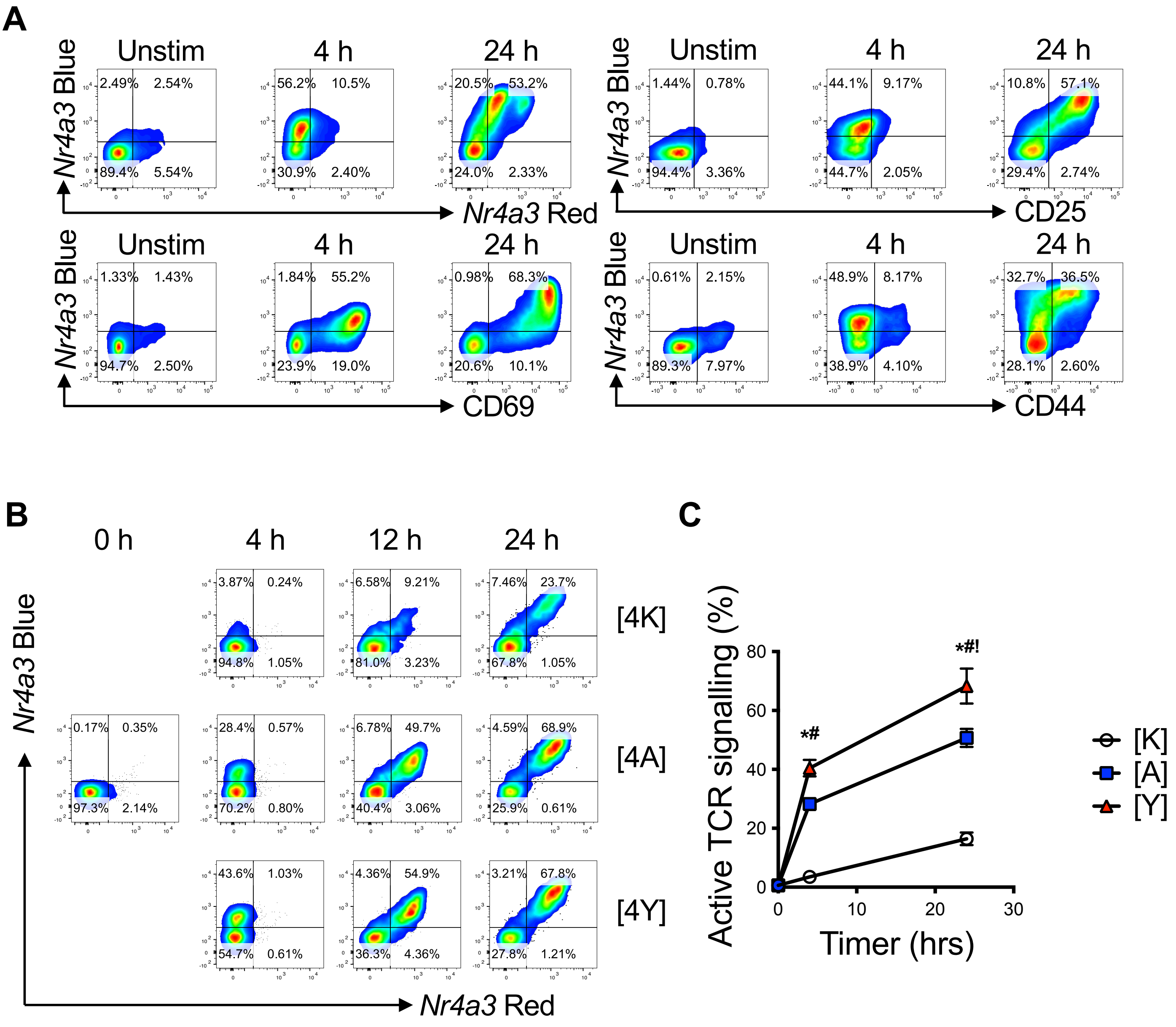

Supplementary Figure 1: Modified MBP peptide variants induce potent T cell activation in vitro (related to Figure 1)

(**A**) Splenocytes from Tg4 *Nr4a3-Tocky Il10*-GFP mice were incubated with 10 μM of [4Y] MBP peptide for the indicated times before analysis of CD4$^+$ Tg4 T cells by flow cytometry for the markers shown. (**B**) Naïve CD4$^+$ T cells from Tg4 *Nr4a3*-Tocky *Il10*-GFP mice were incubated with CD90-depleted splenocytes in the presence of 1 μM of native [4K] MBP peptide, or [4A] or [4Y] variants for the times indicated before analysis of *Nr4a3*-Blue vs *Nr4a3*-Red expression in CD4$^+$ Tg4 T cells. (**C**) Summary data showing the % *Nr4a3*-Blue$^+$ in CD4$^+$ T cells in the 3 peptide groups. MBP [4K] = white circles, MBP [4A] = black squares and MBP [4Y] = red circles. Bars represent mean ±SEM, n=3. Statistical analysis by two-way ANOVA with Tukey's multiple comparisons test. Significant differences between [4Y] and [4K] = *, [4A] and [4K] = #, or [4Y] and [4A] = !.

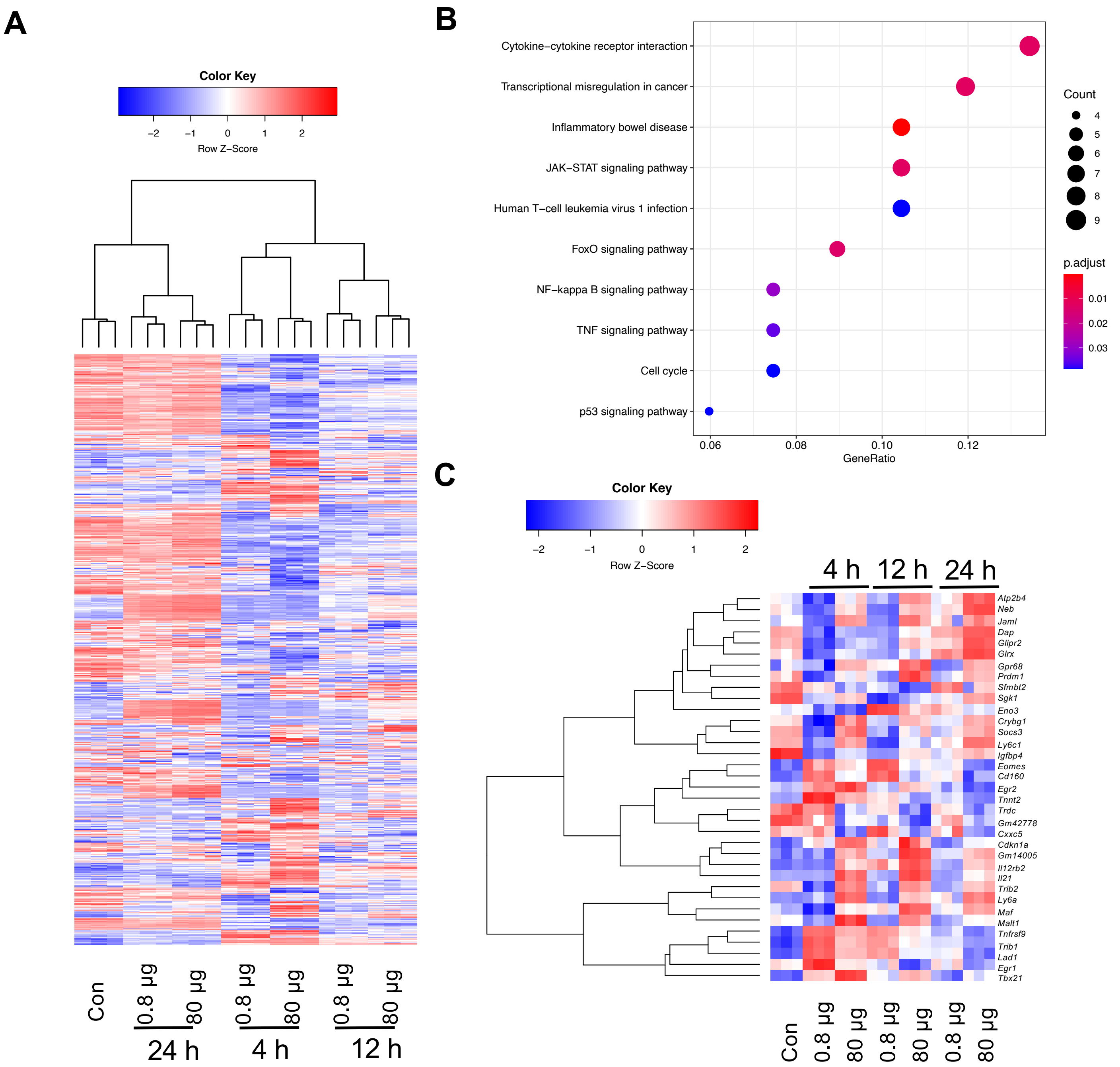

Supplementary Figure 2: Analysis of DEGs in T cells receiving strong or weak TCR signalling in vivo (related to Figure 2)

(A) Z-score heatmap analysis of log2 transformed and normalised counts for all unique DEG identified between 0.8 and 80 µg groups at 4, 12 or 24 h. (**B**) KEGG pathway analysis of DEG at 24 h between 0.8 µg and 80 µg [4Y] MBP immunised mice. (**C**) Z-score heatmap analysis of log2 transformed and normalised counts for genes that show differential expression across all 3 time points (4, 12 and 24 h) analysed between 0.8 µg and 80 µg immunised groups.

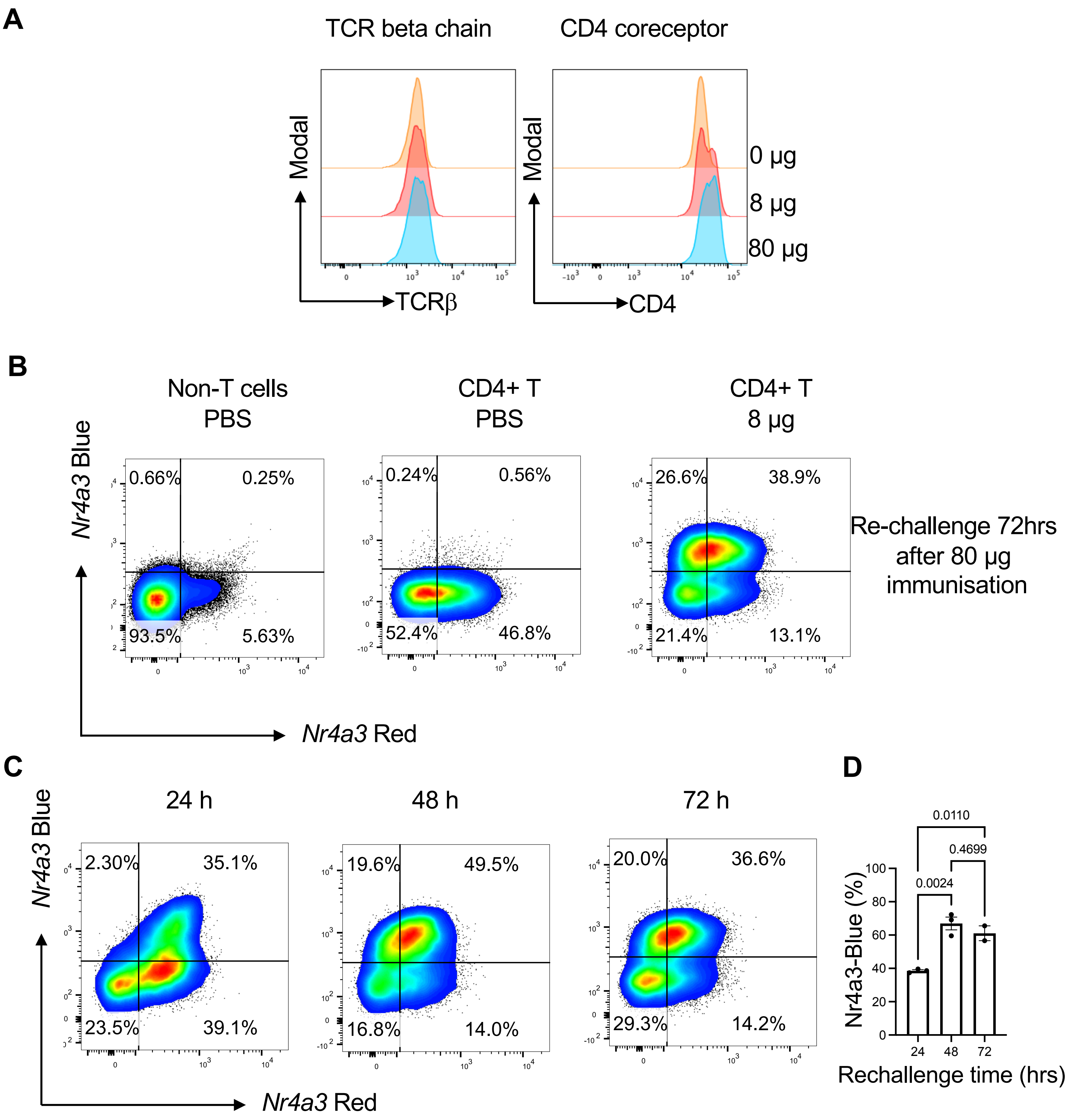

Supplementary Figure 3: Reduced *Nr4a3* activation is not related to TCR levels but is affected by the time between stimulations (related to Figure 4)

(A) Tg4 *Nr4a3*-Tocky *Il10*-GFP mice were immunised s.c. with 0 µg, 8 µg or 80 µg of [4Y] MBP peptide for 24 h. Mice were then rechallenged with 8 µg [4Y] MBP for four h before splenic CD4⁺ *Nr4a3*-Timer⁺ T cells were analysed for their expression of TCRβ and CD4. (**B**) Tg4 *Nr4a3*-Tocky *Il10*-GFP mice were immunised s.c. with 80 µg [4Y] MBP. 72 h later mice were rechallenged with 8 µg or PBS and then splenic CD4⁺ T cell responses analysed for *Nr4a3*-Blue vs *Nr4a3*-Red expression. (**C**) Tg4 *Nr4a3*-Tocky *Il10*-GFP mice were immunised s.c. with 80 µg of [4Y] MBP. Mice were then rechallenged at the indicated time points with 8 µg [4Y] MBP and analysed 4 h later for *Nr4a3*-Blue vs *Nr4a3*-Red expression. (D) Summary data from (C). Bars represent mean ± SEM, statistical analysis by one-way Anova, with Tukey's multiple comparisons test.

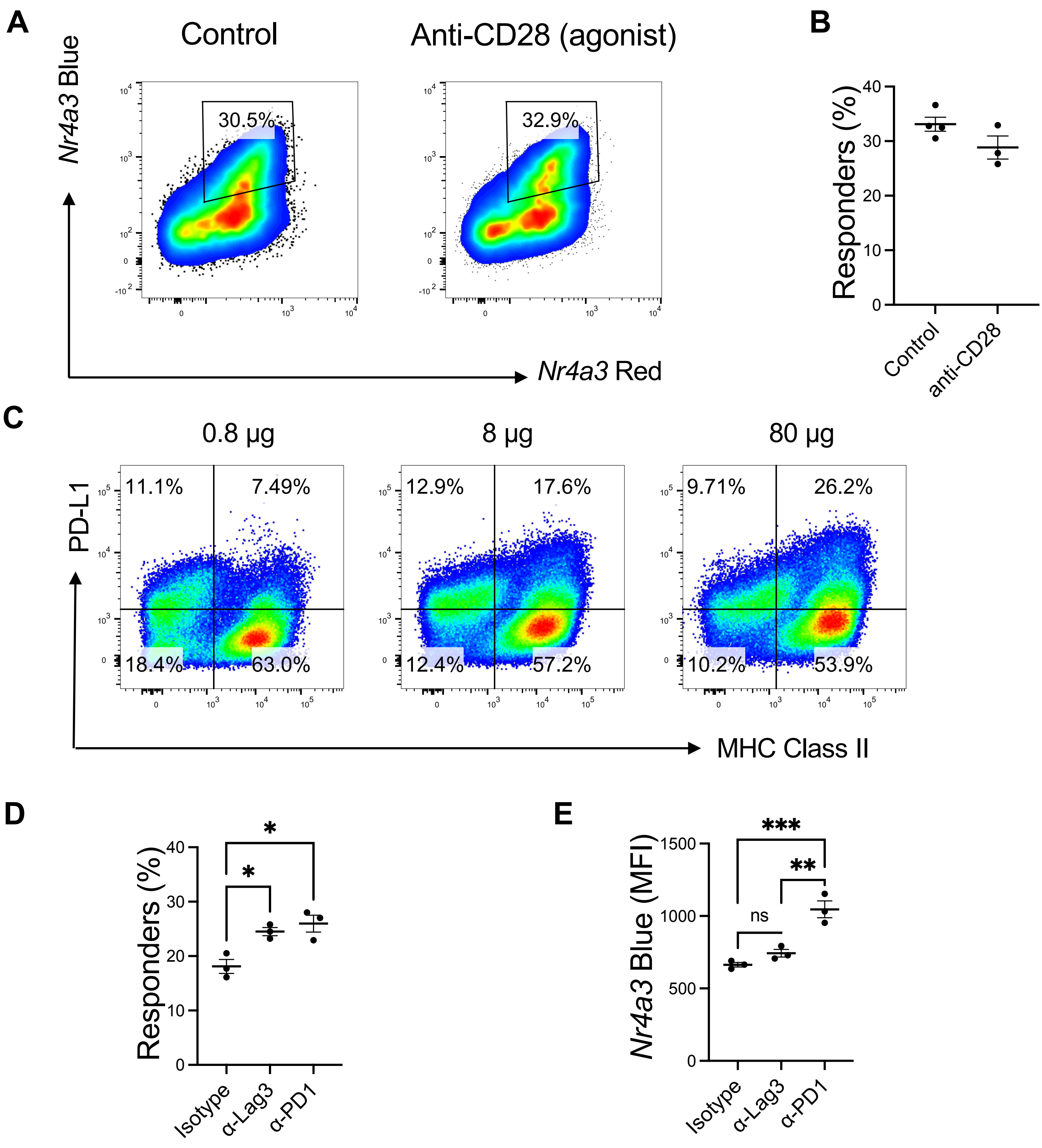

Supplementary Figure 4: Effects of CD28, Lag3 and PD1 pathways on T cell re-activation in vivo (related to Figure 5)

(**A**) Tg4 *Nr4a3*-Tocky *Il10*-GFP mice were immunised s.c. with 80 µg of [4Y] MBP. 24 h later mice were randomised to receive either PBS or 0.5 mg agonistic anti-CD28 30 minutes prior to re-challenge with 8 µg [4Y] MBP peptide. Splenic CD4+ T cells were analysed for *Nr4a3*-Blue vs. *Nr4a3*-Red analysis 4 h after peptide rechallenge. (**B**) Summary data of (**A**), control n=4, anti-CD28 n=3. (**C**) Tg4 *Nr4a3*-Tocky *Il10*-GFP mice were immunised s.c. with 0 µg, 8 µg or 80 µg for 24 h before analysis of live splenocytes for MHC Class II versus PD-L1 expression. (**D&E**) Tg4 *Nr4a3*-Tocky *Il10*-GFP mice were immunised s.c. with 80 µg of [4Y] MBP. 24 h later mice were randomised to receive either isotype, anti-Lag3 or anti-PD1 30 minutes prior to re-challenge with 8 µg [4Y] MBP peptide. The frequency of responder (*Nr4a3*-Blue$^+$Red$^+$) T cells (**D**) or *Nr4a3*-Blue Median expression in responder (*Nr4a3*-Blue$^+$Red$^+$) T cells (**E**) 4 h after peptide rechallenge are shown. N=3, dots represent individual mice, bars represent mean ±SEM. Statistical analysis by one-way ANOVA with Tukey's multiple comparisons test. *=p<0.05, **=p<0.01, ***=p<0.001.

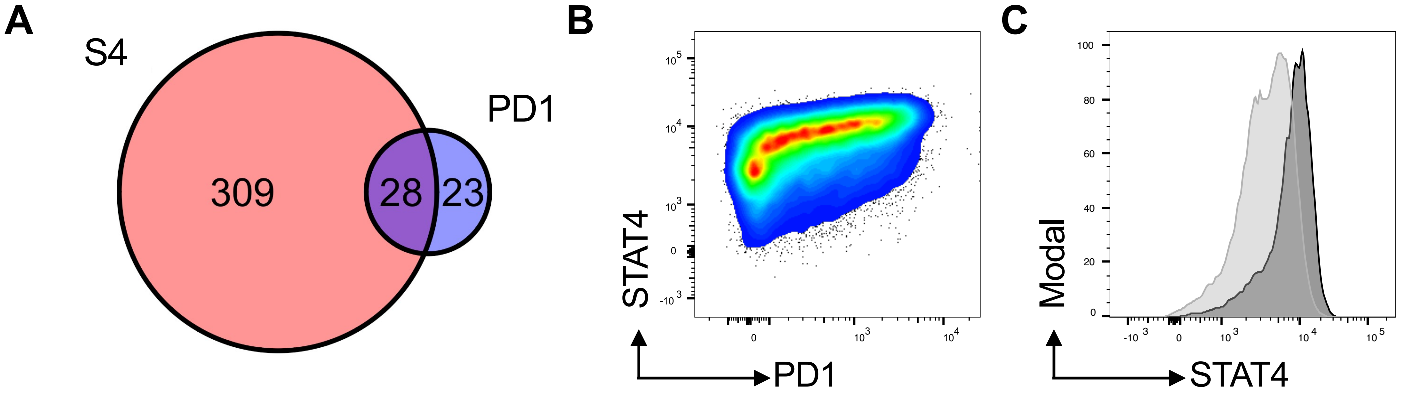

Supplementary Figure 5: Majority of anti-PD1 specific T cell genes are upregulated in T cells receiving strong TCR signal, and STAT4 is increased in activated T cells (related to Figures 2, 5 and 6)

(**A**) Genes upregulated in Tg4 CD4^{+} T cells 4 h after receiving 80 µg vs 0.8 µg [4Y] MBP (Figure 2) were intersected with genes selectively upregulated at 4hrs of T cells re-activated in the presence of anti-PD1 in vivo (Figure 4). 28 out of 51 genes were overlapping and utilised to interrogate MC38 tumour responses in Figure 6. (**B**) Tg4 *Nr4a3*-Tocky *Il10*-GFP mice were immunised s.c. with 80 µg of [4Y] MBP. 24 h later mice received 0.5 mg rat IgG2a 30 minutes prior to re-challenge with 8 µg [4Y] MBP peptide. 4 h later intracellular STAT4 and PD1 expression was analysed in splenic CD4^{+} T cells. (**C**) STAT4 levels in PD1^{hi} (black histogram) or PD1^{lo} (grey histogram) from CD4^{+} T cells in (**B**).

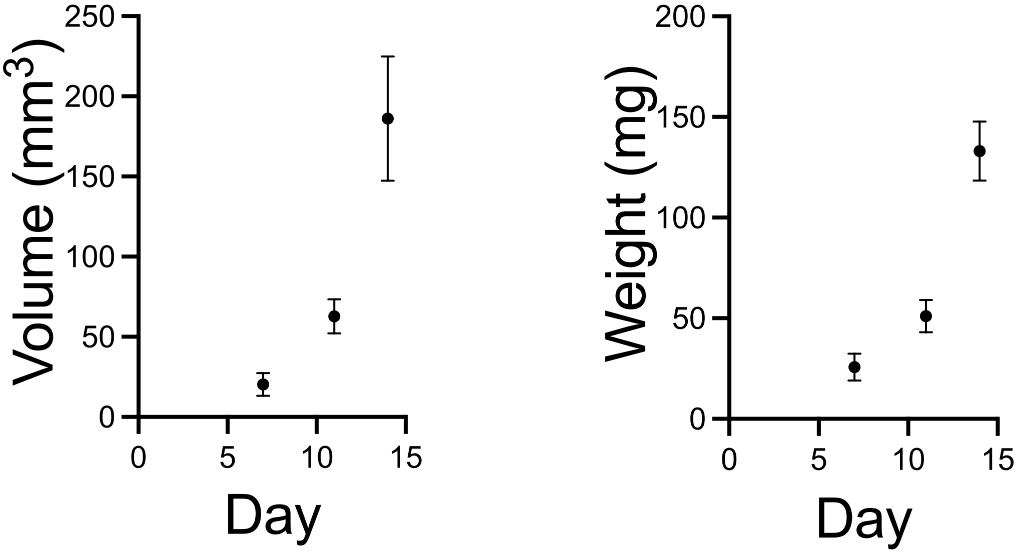

Supplementary Figure 6: MC38 tumour volume and weight (related to Figure 6)

Nr4a3-Tocky *Ifng*-YFP mice were injected with 0.25 M MC38 cells and tumours measured with callipers at the indicated times or weighed upon sacrificing of the mice. N=3, bars represent mean ±SEM.

A

TCR.strong genes
TNFRSF4, ICOS, IRF8,
TNIP3, STAT4

T activation genes
CD69, IL2RA, NR4A1, TNFRSF9

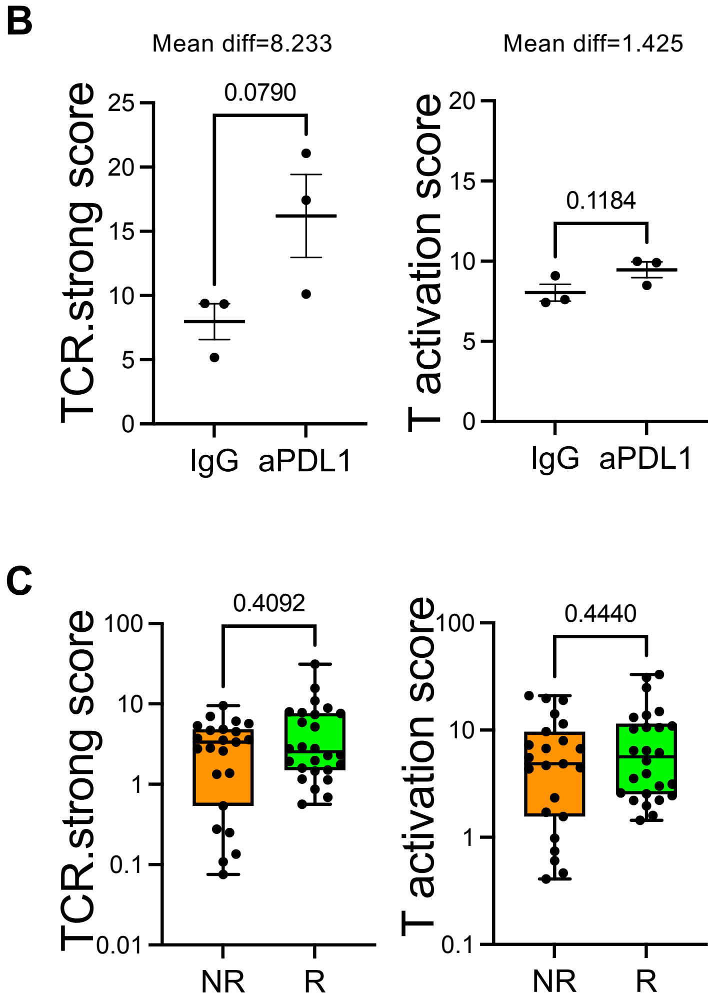

Supplementary Figure 7: TCR.strong and T activation metric analysis (related to Figure 7)

(A) Genes included in the TCR.strong and T activation gene metrics. (**B**) TCR.strong and T activation scores for mice in Figure 6 E&F from (Efremova et al., 2018). Statistical analysis by student t test. (C) Analysis of pre-therapy TCR.strong (left) or T activation (right) scores in patients from the Riaz et al. cohort divided into non responder (NR, orange) and responder (R, green). Dots represent individual patients. Statistical analysis by Mann Whitney U test.
