# Supplementary material for: Antigen and checkpoint receptor engagement recalibrates T cell receptor signal strength
Source: Immunity. 2021 Nov 9;54(11):2481–2496.e6. doi: 10.1016/j.immuni.2021.08.020 (PMC8585507; doi:10.1016/j.immuni.2021.08.020)
Supplement: Document S2. Article plus supplemental information [file mmc8.pdf]

Immunity

Article

Antigen and checkpoint receptor engagement recalibrates T cell receptor signal strength

Graphical abstract

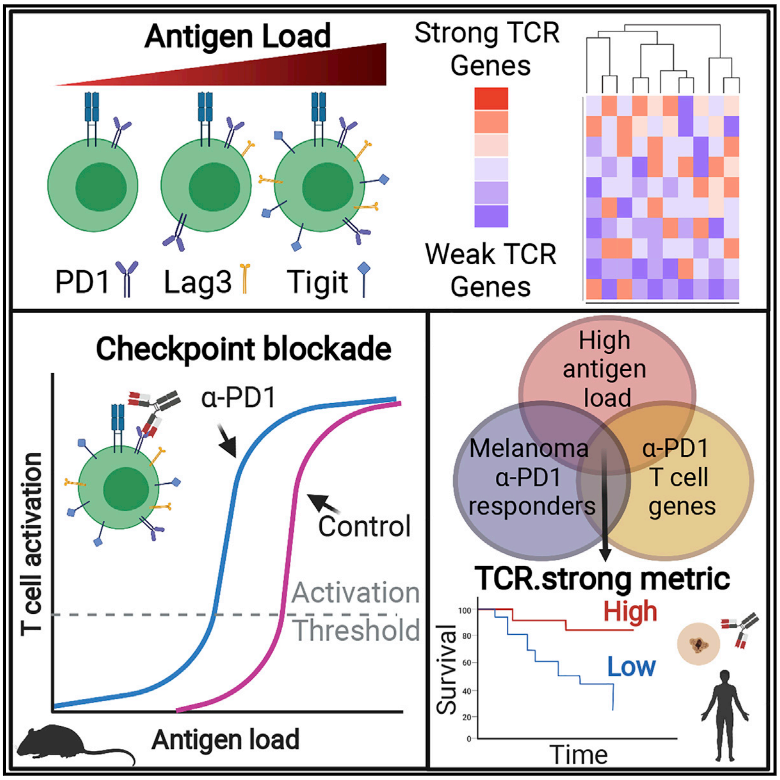

Highlights

- TCR signal strength drives dynamic and time-dependent changes in $CD4^+$ T cells
- Inhibitory receptor expression recalibrates T cell activation thresholds
- PD1 blockade leads to a strong TCR signal signature in T cells (TCR.strong)
- TCR.strong can stratify melanoma patient responses to anti-PD1 therapy

Authors

Thomas A.E. Elliot, Emma K. Jennings, David A.J. Lecky, ..., Kendle M. Maslowski, David C. Wraith, David Bending

Correspondence

d.a.bending@bham.ac.uk

In brief

How antigen and immune checkpoint engagement regulate T cell function is not completely understood. Elliot et al. reveal how antigen abundance regulates immune checkpoint expression and recalibrates T cell activation thresholds. PD1 blockade lowers the T cell activation threshold, resulting in a transcriptional signature that stratifies responses to immunotherapy.

Elliot et al., 2021, Immunity *54*, 2481–2496
November 9, 2021 © 2021 The Author(s). Published by Elsevier Inc.
https://doi.org/10.1016/j.immuni.2021.08.020

Immunity

CellPress
OPEN ACCESS

Article

Antigen and checkpoint receptor engagement recalibrates T cell receptor signal strength

Thomas A.E. Elliot,[1] Emma K. Jennings,[1,4] David A.J. Lecky,[1] Natasha Thawait,[1,5] Adriana Flores-Langarica,[2] Alastair Copland,[1] Kendle M. Maslowski,[1,3] David C. Wraith,[1] and David Bending[1,6,7,*]

[1]Institute of Immunology and Immunotherapy, College of Medical and Dental Sciences, University of Birmingham, Birmingham B15 2TT, UK

[2]Infrastructure and Facilities, College of Medical and Dental Sciences, University of Birmingham, Birmingham B15 2TT, UK

[3]Institute of Metabolism and Systems Research, College of Medical and Dental Sciences, University of Birmingham, Birmingham B15 2TT, UK

[4]Present address: Leeds Institute for Medical Research, Faculty of Medicine and Health, University of Leeds, St James' University Hospital, Leeds LS9 7TF, UK

[5]Present address: Centre for Inflammation Research, Queen's Medical Research Institute, University of Edinburgh, Edinburgh, EH16 4TJ, UK

[6]Twitter: @BendingLab

[7]Lead contact

*Correspondence: d.a.bending@bham.ac.uk

https://doi.org/10.1016/j.immuni.2021.08.020

SUMMARY

How T cell receptor (TCR) signal strength modulates T cell function and to what extent this is modified by immune checkpoint blockade (ICB) are key questions in immunology. Using *Nr4a3*-Tocky mice, we characterized early quantitative and qualitative changes that occur in CD4$^+$ T cells in relation to TCR signaling strength. We captured how dose- and time-dependent programming of distinct co-inhibitory receptors rapidly recalibrates T cell activation thresholds and visualized the immediate effects of ICB on T cell re-activation. Our findings reveal that anti-PD1 immunotherapy leads to an increased TCR signal strength. We defined a strong TCR signal metric of five genes upregulated by anti-PD1 in T cells (TCR.strong), which was superior to a canonical T cell activation gene signature in stratifying melanoma patient outcomes to anti-PD1 therapy. Our study therefore reveals how analysis of TCR signal strength—and its manipulation—can provide powerful metrics for monitoring outcomes to immunotherapy.

INTRODUCTION

How T cells interpret T cell receptor (TCR) signals to promote different functional programs is a critical aspect of their biology. A key feature of T cell activation is the release of intracellular calcium stores to trigger activation of nuclear factor of activated T cells (NFAT) (Hogan et al., 2003). This process occurs in a digital and probabilistic fashion (Gallagher et al., 2018; Podtschaske et al., 2007). Similar results are reported for extracellular signal-regulated kinase (ERK) activation (Altan-Bonnet and Germain, 2005; Das et al., 2009). Nonetheless, despite these digital behaviors, TCR signal strength can lead to graded expression of molecules such as interferon regulatory factor 4 (IRF4) (Conley et al., 2020), Nr4a1 (Moran et al., 2011), and co-inhibitory receptors (Trefzer et al., 2021). Reduced TCR signal strength can also drive graded nuclear factor kappa B (NF-κB) activation (Gallagher et al., 2020). NF-κB activation plays critical roles in T cell activation, with the activity of mucosa-associated lymphoid tissue lymphoma translocation protein 1 (Malt1) paracaspase being key to full NF-κB activity and interleukin-2 (IL-2) expression (Rebeaud et al., 2008).

TCR signal strength does not influence CD8$^+$ T cell end-stage cytolytic capacity *in vitro* (Richard et al., 2018). However, analysis of thymic CD4$^+$ T cell development clearly demonstrates that strong and persistent TCR signals drive Foxp3$^+$ regulatory T (Treg) cell development (Bending et al., 2018b; Jennings et al., 2020; Moran et al., 2011), and antigen affinity and dose have distinct effects on peripheral CD4$^+$ T cells (Keck et al., 2014; Trefzer et al., 2021). Understanding how graded responses to TCR signal strength can modulate T cell function will likely be critical to understanding mechanisms behind immunotherapies. For example, key T cell transcripts may require differing thresholds of TCR signal strength (Shimizu et al., 2020).

While many studies investigate TCR signaling using *in vitro* systems, the study of how TCR signal strength regulates T cell activation, and how immunotherapy may alter these processes, is far from clear. Antigen concentration influences the rates of T cell activation, meaning *in vivo* studies may struggle to dissect differences that occur because of differing T cell activation kinetics (Richard et al., 2018). Furthermore, different T cell genes require different durations of TCR signals for expression (Jennings et al., 2020).

To address the challenges of studying T cell activation dynamics, we previously developed the *Nr4a3*-Timer of cell kinetics and activity (Tocky) model (Bending et al., 2018b). *Nr4a3*-Tocky mice are NFAT-responsive distal TCR signaling

This is an open access article under the CC BY license (http://creativecommons.org/licenses/by/4.0/).

reporter mice (Jennings et al., 2020; Jennings et al., 2021). *Nr4a3*-Tocky utilizes a fluorescent timer protein (Subach et al., 2009) to monitor the temporal dynamics of TCR signaling and can classify TCR signals according to whether they are *new*, *persistent*, or *arrested* (i.e., TCR signaling was initiated and has now recently stopped). Given that NFAT is necessary and sufficient for expression of *Nr4a3* in T cells (Jennings et al., 2020; Martinez et al., 2015), we predicted that *Nr4a3* would represent a digital readout for T cell activation *in vivo*, which would permit the tracking of T cells following antigen encounter over the first 24 h. Here, we employed the Tg4 *Nr4a3*-Tocky (Jennings et al., 2020) mouse model to track synchronized T cell activation *in vivo* and identify quantitative and qualitative changes that occur in T cells receiving different strengths of TCR signaling *in vivo*. Crucially, this system accounted for differing proportions of T cells that may respond while also permitting the analysis of T cells at different synchronized phases following T cell activation. Our findings identified the relationships between TCR signal strength and key T cell transcriptional programs, including the programming of temporally distinct co-inhibitory receptor modules. These modules rapidly recalibrated the activation threshold of T cells, allowing direct detection of the immediate effects of immune checkpoint blockade on T cell reactivation *in vivo*. We refined a TCR signal strength metric down to 5 genes specifically upregulated by anti-PD1 in T cells (called TCR.strong), which stratifies clinical outcomes following anti-PD1 therapy in melanoma patients.

RESULTS

Antigen dose drives digital *Nr4a3* activation at the single-cell level but graded responses at population and phenotypic levels

We crossed the *Nr4a3*-Tocky system (Jennings et al., 2020) with the Tg4 TCR transgenic line that recognizes myelin basic protein (MBP) peptide (Figure 1A). This system has been useful in assessing the response of T cells to modified self-antigens under tolerogenic immunizing conditions, and it is known that repeated dosing of this system imparts a type 1 regulatory (Tr1) T cell phenotype (Burton et al., 2014) supported by epigenetic remodeling (Bevington et al., 2020). To monitor changes in *Il10* expression, we incorporated an *Il10*-IRES-GFP reporter (Kamanaka et al., 2006). *In vitro* experiments demonstrated the correlative relationship between *Nr4a3*, CD69, CD25, and CD44 expression (Figure S1A). Activation with the native lysine at position 4 [4K] MBP peptide induced weak activation of Tg4 T cells (Figures S1B and S1C). Switching of the fourth peptide residue to alanine [4A] or tyrosine [4Y] increased the potency of TCR signaling (Figures S1B and S1C).

To determine how TCR signal strength affected NFAT-*Nr4a3* activation *in vivo*, a hundred-fold range of [4Y] MBP peptide was administered subcutaneously (s.c.) (without adjuvant under tolerising conditions) to Tg4 *Nr4a3*-Tocky *Il10*-GFP mice (Figures 1B–1D). At 4 h, splenic T cells responded with an increase in *Nr4a3*-Blue, indicating new TCR signaling in response to recognition of the [4Y] MBP peptide. By 12 h, a population of CD4$^+$ T cells were *Nr4a3*-Blue$^+$Red$^+$, indicating the increased time elapsed since initiation of TCR signaling. By 16–24 h, responding T cells had migrated toward the *arrested* *Nr4a3*-Timer locus (Figure 1B) (Bending et al., 2018b). The arrested locus defines cells that have recently terminated the TCR signal but retain red fluorescence because of its longer half-life than blue. This indicated that most T cells experiencing stimulation in this model arrest *Nr4a3* expression within the first 24 h. Analysis of active TCR signaling (i.e., all *Nr4a3*-Blue$^+$ cells) showed a peak at 4 h, before a fall to near-zero by 24 h (Figure 1C). These results show that the proportion of responding T cells was dependent on TCR signal strength. However, analysis of *Nr4a3*-Timer Angle (which determines the average position of *Nr4a3*-Timer$^+$ T cells in blue-red space; Bending et al., 2018b) showed highly similar Timer trajectories independent of the immunizing dose (Figure 1D). Therefore, the strength of TCR signaling did not affect the dynamics of *Nr4a3* activation; moreover, at the single-cell level, those T cells that crossed the threshold of activation of the NFAT-*Nr4a3* pathway exhibited highly similar dynamics of *Nr4a3* expression. However, in the 24 h stimulation period, early expression of *Il10*-GFP emerged within *Nr4a3*-Timer$^+$ T cells with a direct correlation to the amount of immunizing antigen (Figures 1E and 1F), reflecting that TCR signal strength can impart rapid phenotypic heterogeneity within activated T cell populations *in vivo*.

CD4$^+$ T cells rapidly discriminate stimulation strength through transcriptionally distinct and time-dependent activation profiles

Based on the link between *Il10*-GFP and TCR signal strength, we hypothesized that TCR signal strength controls the proportion of activated T cells and phenotypically distinct activation profiles. We repeated *in vivo* s.c. immunizations of *Nr4a3*-Timer Tg4 Tiger mice with [4Y] MBP peptide at a 100-fold dose range (Figure 2A). In order to control for quantitative differences between the two conditions, we sorted cells based on their Timer protein maturation (Figure 2A). This allowed us to isolate T cell populations from different conditions at highly synchronized stages of TCR signaling. RNA was extracted from sorted cells, and 3′ mRNA libraries were prepared for RNA sequencing (RNA-seq). Principal component analysis (PCA) revealed four clusters (Figure 2B). Within each time cluster, they separated into two distinct groups based on the amount of immunizing antigen. We focused our analysis on differentially expressed genes (DEGs) between the low and high antigen groups (Figure 2C; Table S1). Most DEGs were present at the 4-h time point, which declined over time. Analyzing the DEGS at the different time points suggested that most of these genes were unique to the time point of analysis (Figure 2D). Heatmap analysis of the cumulative DEGs across the 3 time points revealed that 24-h samples clustered tightly with the non-activated control population (Figure S2A), however, the 4- and 12-h clusters separatedinto discrete branches. To understand biological processes that are influenced by TCR signal strength, we performed Kyoto encyclopedia of genes and genomes (KEGG) pathway analysis (Figures 2E and 2F; Table S2). Notable pathways showing enrichment at 4 h included cytokine-cytokine receptor interactions, JAK-STAT signaling, T helper-17 (Th17) cell differentiation, Th1 and Th2 cell differentiation, and TCR signaling pathways (Figure 2E). Several of these pathways were still enriched at 12 h (Figure 2F). Analysis of 24-h DEGs reflected sustained changes in cytokine-cytokine receptor interactions and JAK-STAT signaling (Figure S2B). Based on

CellPress
OPEN ACCESS

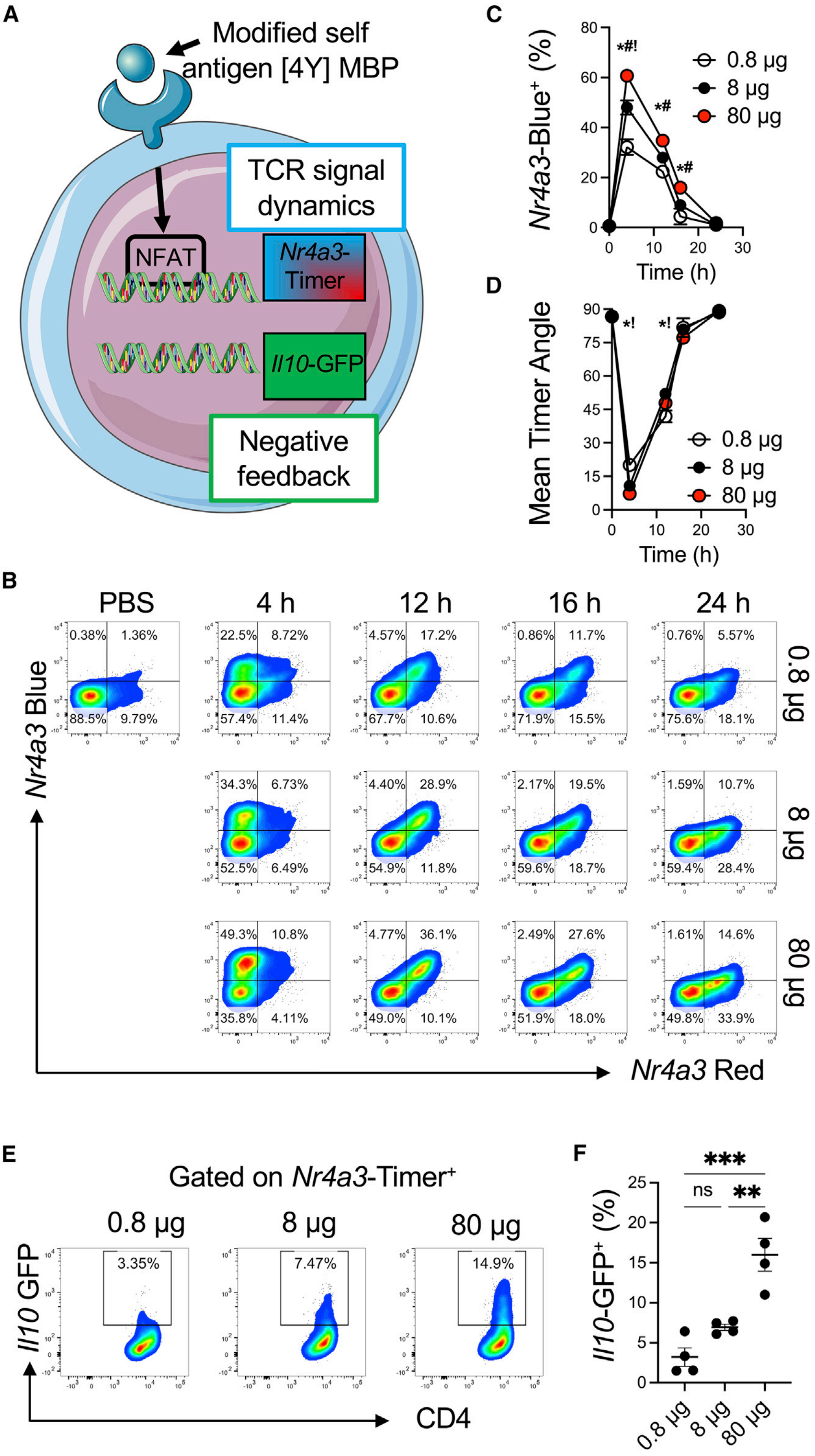

Figure 1. Antigen dose drives digital *Nr4a3* activation at the single-cell level but graded responses at population and phenotypic levels

(A) Tg4 *Nr4a3*-Tocky *Il10*-GFP system.

(B) Tg4 *Nr4a3*-Tocky *Il10*-GFP mice were immunized s.c. with 0.8 μg, 8 μg, or 80 μg of [4Y] MBP peptide (without adjuvant) and splenic CD4$^+$ T cell responses analyzed for *Nr4a3*-Red versus *Nr4a3*-Blue expression in live CD4$^+$ Tg4 T cells.

(C and D) Summary data of (C) the percent of CD4$^+$ Tg4 T cells exhibiting active TCR signaling (percentage of total cells *Nr4a3*-Blue$^+$ irrespective of Red status) or (D) mean *Nr4a3*-Timer Angle in 0.8 μg (white), 8 μg (black), or 80 ug (red) immunized mice. Circles represent mean ± SEM. Statistical analysis by two-way Anova with Tukey's multiple comparisons test. Significant differences ($p < 0.05$) between 80 μg and 0.8 μg (*), 80 μg and 8 μg (#), or 8 μg and 0.8 μg (!).

(E) Tg4 *Nr4a3*-Tocky *Il10*-GFP mice were immunized s.c. with 0.8 μg, 8 μg, or 80 μg of [4Y] MBP peptide and splenic CD4$^+$ T cell responses analyzed for CD4 versus *Il10*-GFP in Nr4a3-Timer$^+$ T cells at 24 h post immunization.

(F) Summary data of *Il10*-GFP expressers (percent of CD4$^+$) in the three experimental groups. n = 4, bars represent mean ± SEM, statistical analysis by one-way Anova with Tukey's multiple comparisons test. ***$p < 0.001$, ** = $p < 0.01$.

Please also see Figure S1.

these findings, we conceptualized 7 key modules that were undergoing time- and dose-dependent transcriptional activation or suppression (Figure 2G): (1) a shared activation module, incorporating *Nr4a1-3* receptors, *Cd69* and *Tnf*. (2) A second group of activated genes that trended to have higher and/or longer expression, including *Tnfrsf4* (OX40), *Cd40lg*, the inhibitory receptor *Pdcd1* (PD1), and IL-2 signaling (*Il2* and *Il2ra*). Included here were *Irf4*, *Irf8*, and *Tbx21* (T-bet). (3) A third module incorporating Th1 cell-associated and T cell effector functions *(Ifng*, *Il12rb2*, *Gzmb)*, which exhibited rapid induction at 4 h in the 80-μg group. This module showed delayed activation in the 0.8-μg group; however, *Gzmb* and *Il12rb2* remained high throughout the 24-h period in the 80-μg group. (4) A fourth module specific to strong TCR signaling was upregulated at 4 and 12 h and largely sustained at 24 h. This included the Th17 cell-associated genes *Rora*, *Rorc*, and *Il21*. In addition, *Malt1*, an enzyme involved in NF-κB signaling, was strongly induced at 4 h and 12 h in 80-μg stimulated group along with the *Mt1* and *Mt2* enzymes involved in zinc bioavailability. (5) The fifth module involved genes undergoing strong and sustained expression that was largely specific to high antigen dose. These included *Ctla4*, *Icos*, and *Maf.* (6) The sixth module identified a regulatory motif that appeared transiently at the 12-h time point in the 80-μg group. This module included *Il10* (echoing the findings in Figure 1E), *Lag3*, *Nfil3*, and

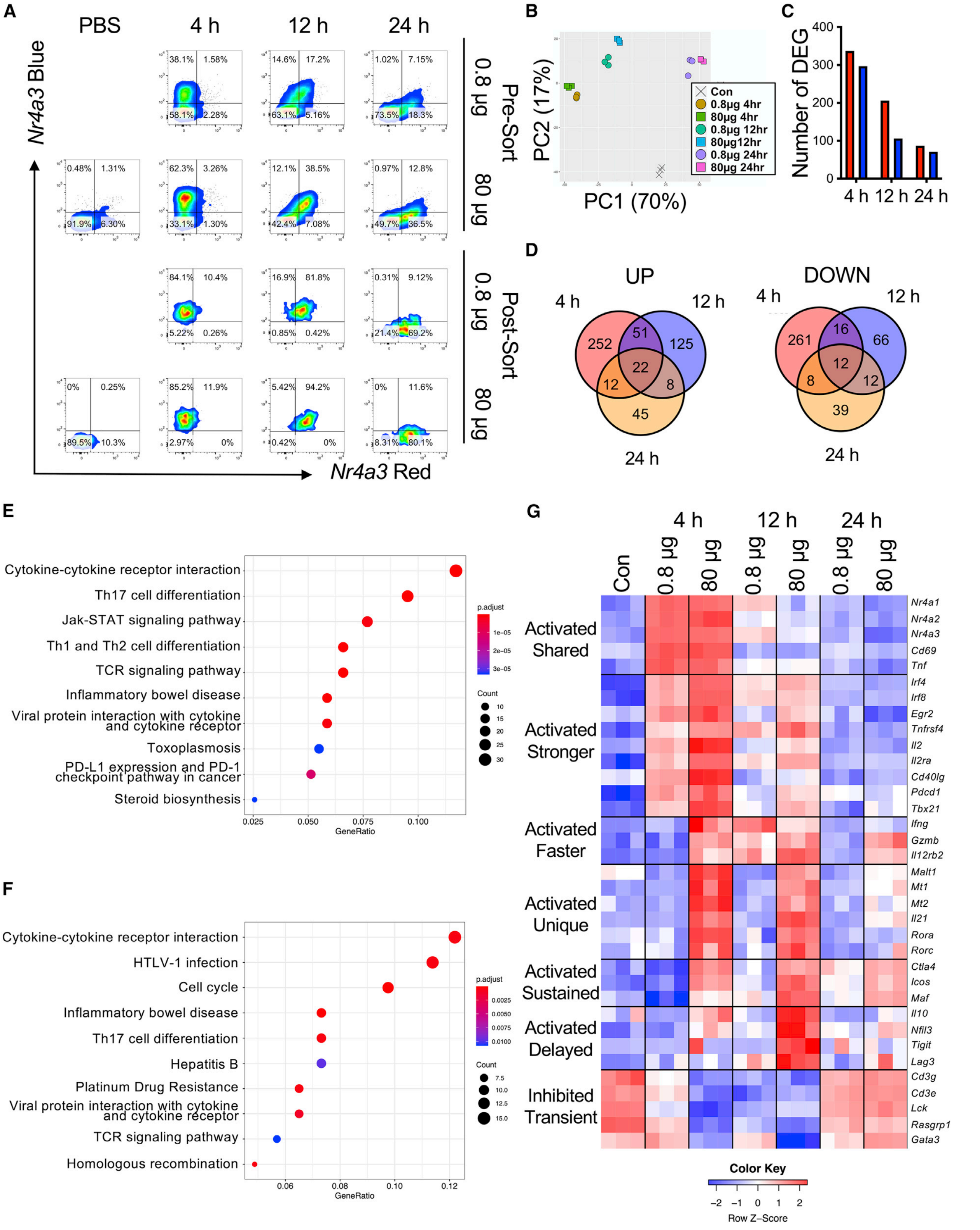

Figure 2. CD4⁺ T cells rapidly discriminate stimulation strength through transcriptionally distinct and time-dependent activation profiles

(A) Tg4 *Nr4a3*-Tocky *Il10*-GFP mice were immunized s.c. with 0.8 μg or 80 μg of [4Y] MBP peptide (without adjuvant) and splenic CD4⁺ T cell responses analyzed for *Nr4a3*-Timer Red versus *Nr4a3*-Timer Blue expression in live CD4⁺ Tg4 T cells at the indicated time points.

(legend continued on next page)

CellPress
OPEN ACCESS

Tigit, which are all associated with Tr1 cells. This module peaked after the termination of TCR signaling in the 80-μg immunized group. (7) A final module exhibited strong but transient downregulation of key parts of the TCR signaling pathway (*Cd3g*, *Cd3e*, *Lck*, *Rasgrp1*) at the 4-h period in the 80-μg group, indicating that negative feedback responses to high antigen dose are stronger in this group; however, by 24 h, the expression of these genes had returned to baseline. Included in this group was the Th2 cell-associated transcription factor *Gata3*, reflecting the KEGG pathway analysis of T cells inducing signatures of Th1 and Th17 cell programs (*Rora*, *Rorc*, *Tbx21*, *Ifng*, *Il12rb2*, *Il21*). Analysis of DEGs across all time points revealed that *Il21*, *Il12rb2*, *Tbx21*, *Maf*, and *Malt1* were sustained across the whole 24-h period, indicating a motif strongly associated with T cells experiencing a very strong TCR signal *in vivo* (Figure S2C). In summary, our analysis identified clear signatures of diverse transcriptional programs being induced in a time- and dose-dependent fashion *in vivo*.

Nr4a3 activation threshold is calibrated by dose-dependent negative feedback

A key finding was the relationship between key negative regulators and TCR signal strength (Figure 3A). PD1 was tightly coupled to T cell activation (Figure 3B) and only modestly influenced by TCR signal strength; Lag3, Tigit, and CTLA-4 (Figures 3C–3E) were very much dependent on the immunizing dose. Given that *Il10*, Lag3, and Tigit appeared as a co-regulated module (Figure 2G), we investigated the notion that *Il10*$^{+}$ T cells reflected those receiving the highest signaling *in vivo*. Analysis of Lag3 and Tigit between *Il10*^{hi} and *Il10*^{lo} populations demonstrated that *Il10*$^{+}$ T cells had significantly higher expression of these receptors (Figures 3F and 3G).

Given the upregulation of multiple immune checkpoints, we hypothesized that T cell responsiveness to acute re-stimulation would be dependent on the immunizing dose. Moreover, we hypothesized that the T cells that arrested TCR signaling in response to weak TCR signaling would be more sensitive to re-stimulation than T cells initially activated with a strong TCR signal (Figure 4A). Because Tg4 *Nr4a3*-Tocky T cells activated with peptide for 24 h move into the Blue^{-}Red^{+} quadrant, due to *arrested* TCR signaling (Figures 1B–1D), re-challenge with peptide at this time point would lead to the re-emergence of *Nr4a3*-Blue expression in this population and move up into the Blue^{+}Red^{+} quadrant. If the re-challenge is analyzed after 4 h, then almost all *Nr4a3*-Blue^{+}Red^{+} T cells will represent T cells that are responding to the first and second stimulations (Figure 4A). This is possible because the half-life of *Nr4a3*-Blue protein is 4 h while *Nr4a3*-Red is 120 h (Bending et al., 2018a). T cells that remain in the lower right quadrant (*Nr4a3*-Red^{+}Blue^{-}) even after re-stimulation would reflect T cells that fail to respond to the second dose. To test this hypothesis, we immunized mice with either 0 μg, 8 μg, or 80 μg of [4Y] MBP to induce no, moderate, or strong TCR stimulation. 24 h later, we sub-divided these three groups into two groups to receive a further 8 μg or 80 μg stimulation for 4 h to trigger *Nr4a3*-Blue expression. Our analysis focused on assessing the proportion of T cells that remained within the *arrested* TCR signaling quadrant (i.e., *Nr4a3*-Blue^{-}Red^{+}). Administration of 0 μg followed by 8 μg or 80 μg induced cells predominantly in the *new* Timer locus (Figure 4B, left). Immunizing with an initial 8 μg and then re-challenge with 8 μg or 80 μg induced a clear *Nr4a3*-Blue^{+}Red^{+}, indicating that the majority of these previously activated T cells responded to the second dose (Figure 4B, middle). In contrast, most T cells immunized with 80 μg and challenged with 8 μg remained in the *arrested* locus (Figure 4B, right). Even when re-stimulating with 80 μg in this group, a proportion of *arrested* TCR signaling cells remained. More T cells failed to reactivate *Nr4a3* expression in response to a second restimulation with 8 μg or 80 μg when the T cells had been first immunized with 80 μg (Figure 4C). This defect was not linked to any differences in the expression of TCR or CD4 (Figure S3A) but was influenced by the timing of the restimulation (Figures S3B–S3D). As predicted by the increased expression of ICBs, *Il10*$^{+}$ T cells showed increased non-responsiveness to re-stimulation compared with *Il10*$^{-}$ counterparts (Figure 4D-E). In summary, our data reveal that T cell activation thresholds are temporarily recalibrated by the initial TCR signaling episode *in vivo* and display heterogeneity in their responsiveness to re-stimulation.

Co-inhibitory receptors exert distinct quantitative and qualitative control over T cell re-activation

We next explored the extent to which different checkpoints could modulate the thresholds for re-activation of T cells *in vivo*. We chose PD1, CTLA-4, and Lag3 pathways to compare checkpoints from the modules identified in Figure 3A. We adapted the model from Figure 4A to include administration of a blocking antibody to the co-inhibitory receptor 30 min before peptide challenge (Figure 5A). Agonistic CD28 antibody did not alter the threshold for activation of T cells in this model (Figures S4A and S4B), so we focused our exploration on the potential roles Lag3 and PD1 play in modulating T cell re-activation (Figure 5B). We confirmed that ligands for the respective co-inhibitory receptors major histocompatibility complex (MHC) class II and PD-L1 were expressed in the splenic environment (Figure S4C). Anti-PD1 blockade induced an increase in responders (Figures 5B–5D), with anti-Lag3 inducing an intermediate effect on the re-activation of T cells. Anti-PD1 induced higher amounts of *Nr4a3*-Blue in responding T cells (Figure 5D) than mediated by the isotype group or anti-Lag3. These data support that PD1 quantitatively controls the activation thresholds of T cells *in vivo* as reported by *Nr4a3* activity.

As anti-PD1 induced higher amounts of *Nr4a3*-Blue, we hypothesized that anti-PD1 may induce qualitative changes within

(B) RNA was extracted from the sorted populations and 3′ mRNA sequencing performed. PCA of the normalized expression data identifies 7 clusters.

(C) Differentially expressed genes (DEGs) identified using DESeq2 between 80 μg and 0.8 μg stimulated T cells at indicated time points. Up DEG are in red and down DEG in blue.

(D) Venn diagram analysis of up and down DEG at 4-, 12-, and 24-h time points.

(E and F) KEGG pathway analysis of DEG between 80 μg and 0.8 μg at 4-h (E) or 12-h (F) time points.

(G) Z score heatmap analysis of log2 transformed and normalized counts.

Please also see Figure S2 and Tables S1 and S2.

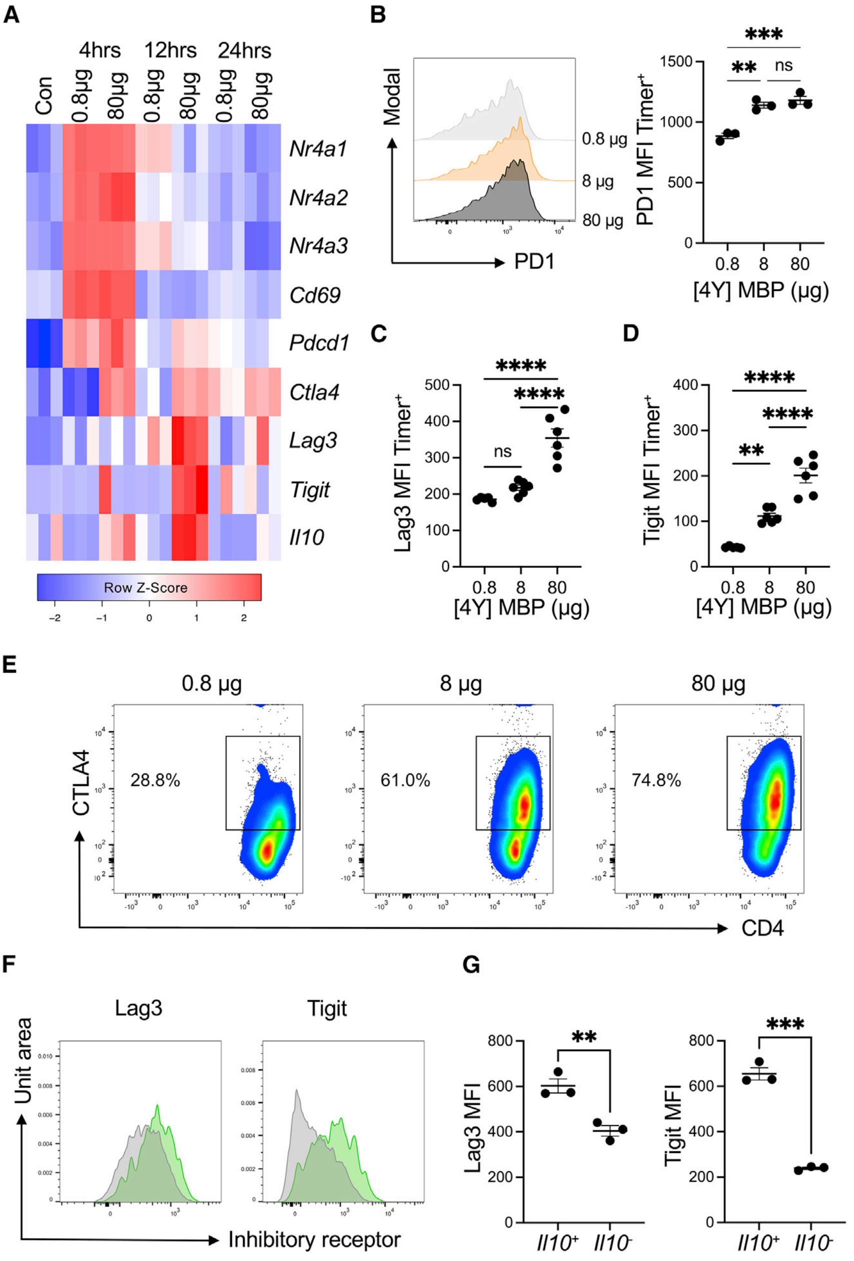

Figure 3. Strong TCR signaling drives high amounts of immune checkpoint expression

(A) Heatmap comparing key inhibitory receptors and their relationships to *Nr4a* expression from Figure 2G.

(B) PD1 expression on live CD4+ *Nr4a3*-Timer+ T cells 12 h following immunization, n = 3. Bars represent mean ± SEM. Statistical analysis by one-way Anova with Tukey's multiple comparisons test. **p < 0.01, ***p < 0.001; ns, not significant.

(C and D) Lag3 (C, n = 6) or Tigit (D, n = 6) expression on live CD4+ *Nr4a3*-Timer+ T cells 24 h after immunization. Statistical analysis by one-way Anova with Tukey's multiple comparisons test. Bars represent mean ± SEM. **p < 0.01, ****p < 0.0001; ns, not significant.

(E) CD4 versus intracellular CTLA-4 expression in live CD4+ T cells 24 h after immunization with the stated doses.

(F) Lag3 and Tigit expression on live CD4+ *Il10*-GFP^{hi} (green) or *Il10*-GFP^{lo} (gray) *Nr4a3*-Timer+ T cells 24 h after immunization with 80 μg [4Y] MBP.

(G) Summary data of (F), n = 3. Statistical analysis by unpaired t test. Bars represent mean ± SEM. **p < 0.01, ***p < 0.001

T cells re-activating in the presence of its blockade. To compare T cells responding to ICB *in vivo*, we isolated *Nr4a3*-Blue+Red+ responder T cells from isotype-, anti-Lag3-, or anti-PD1-treated mice (Figure 5E). We isolated *Nr4a3*-Blue+Red+ to control for differences in the proportions of responding T cells. This experiment once again re-capitulated the quantitative effects of PD1 and Lag3 blockade on the frequency of responding cells (Figures S4D and S4E). RNA was extracted from these sorted T cells and subjected to 3′ mRNA sequencing. PCA analysis showed that anti-PD1 T cells clustered as a separate group to the isotype and anti-Lag3 groups (Figure 5F). 69 DEGs existed between the anti-PD1 and isotype group, demonstrating that 4 h of T cell activation in the presence of anti-PD1 is sufficient to impart qualitative changes within responding T cell populations (Table S3). KEGG pathway analysis revealed signatures very similar to those observed in the strong TCR signaling analysis in Figures 2E and 2F. Cytokine-cytokine receptor, JAK-STAT, Th1, Th2, Th17 differentiation, TCR signaling, PD-L1 expression, and PD-1 checkpoint pathway in cancer were enriched terms (Figure 5G). Heatmap analysis showed anti-PD1 clustered distinct from isotype or anti-Lag3 groups (Figure 5H). Notably, we saw an enrichment of costimulatory receptors, including *Tnfrsf4*, *Tnfrsf9*, *Tnfrsf18*, and *Icos*. In addition to *Irf4* and *Irf8*, as with 80 μg versus 0.8 μg primary TCR stimulation (Figure 2), *Il21*, *Il12rb2*, and *Malt1* were also upregulated in anti-PD1-treated T cells (as had been identified as sustained markers in Figure 2G). Given the similarity between the genes upregulated in the anti-PD1 group compared with controls and those identified in T cells stimulated for 4 h with a high antigen dose, we compared the intersect of the DEGs between the two mRNA-seq experiments (Figure S5A). Our analysis showed that 28 out of 51 of the genes upregulated in T cells re-activated in the presence of anti-PD1 were also genes upregulated in T cells experiencing a strong initial TCR signal for 4 h (Figure S5A). Protein analysis of notable gene members showed that OX40, GITR, and IRF8, but not ICOS, were increased in tandem with *Nr4a3*-Blue 4 h after re-challenge of Tg4 *Nr4a3*-Tocky T cells (Figures 5I–5M). While STAT4 was increased on PD1+ T cells (Figures S5B and S5C), at the 4 h stage, no significant differences between STAT4 were observed between isotype- or

CellPress
OPEN ACCESS

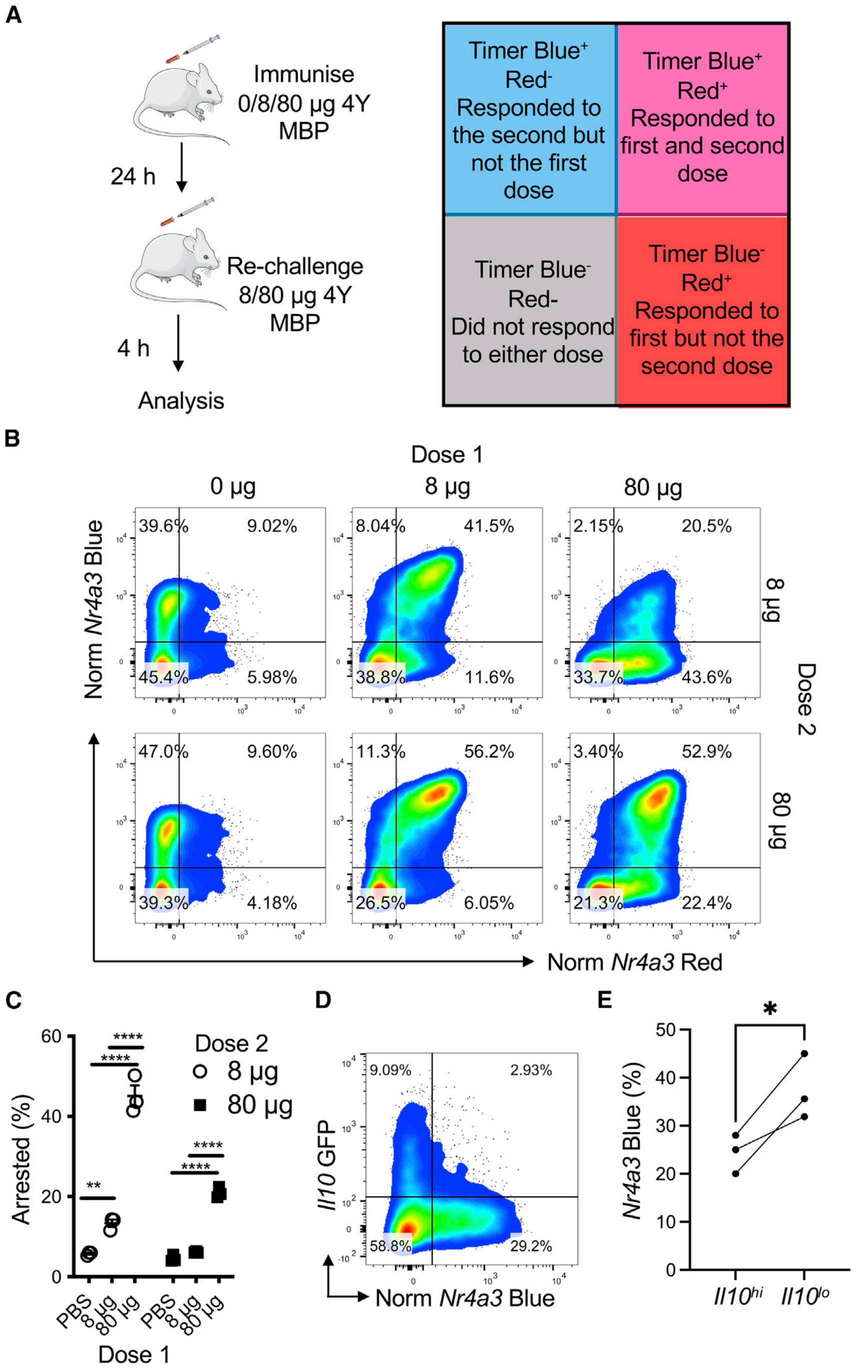

Figure 4. *Nr4a3* activation threshold is calibrated by dose dependent negative feedback

(A) Experimental setup and interpretation for part (B).

(B) Tg4 *Nr4a3*-Tocky *Il10*-GFP mice were immunized s.c. with 0 μg, 8 μg, or 80 μg of [4Y] MBP. 24 h later mice were randomized to receive either 8 μg or 80 μg [4Y] MBP re-challenge before splenic CD4^{+} T cells were analyzed for normalized *Nr4a3*-Timer Blue versus normalized *Nr4a3*-Timer Red analysis 4 h after peptide re-challenge.

(C) Summary data of the frequency of *arrested* TCR signaling T cells from (B), n = 3, bars represent mean ± SEM, statistical analysis by two-way Anova with Sidak's multiple comparisons test.

(D) Tg4 *Nr4a3*-Tocky *Il10*-GFP mice were immunized for 24 h with 80 μg [4Y] MBP before re-challenge for 4 h with 8 μg [4Y] MBP and then normalized *Nr4a3*-Timer Blue versus *Il10*-GFP analyzed in CD4^{+} Tg4 T cells.

(E) Summary data of percent of *Nr4a3*-Blue^{+} following 8 μg re-challenge in (D) in *Il10*-GFP^{hi} versus *Il10*-GFP^{lo} cells, n = 3. Statistical analysis by paired t test.

Please also see Figure S3.

anti-PD1-treated mice (Figure 5N). These data support our hypothesis that anti-PD1 not only increases the probability of a T cell re-activating *Nr4a3* because of lowering the threshold of activation, but also results in a qualitatively stronger TCR signal.

Strong TCR signaling genes are induced in tumors by anti-PD-L1 treatment

To investigate the relevance of our T cell gene signatures further, we utilized the MC38 colorectal cell line model. Our aim was to test whether the effects of our signature could be found across a whole-tumor biopsy landscape *in vivo*. Although our signature was identified in CD4^{+} T cells, we took an agnostic approach to the relative contribution of T cell subsets to tumor immunity. While CD8^{+} T cell function (such as cytolytic capacity; Rooney et al., 2015) are well established, CD4^{+} T cell help is important for CD8^{+} T cell responses in cancer (Borst et al., 2018) and both subsets are required in syngeneic tumor models in mice for anti-PD1 pathway responsiveness (Homet Moreno et al., 2016). Furthermore, like CD8^{+} T cells, CD4^{+} T cells can also act in a cytolytic fashion in human cancer (Cachot et al., 2021).

We injected MC38 tumor cells into the flanks of *Nr4a3*-Tocky *Ifng*-YFP mice to examine the dynamics of tumor development and T cell responses. Tumors increased in weight and volume from day 7 to day 14 (Figure S6). At days 11 and 14, CD4^{+} and CD8^{+} tumor-infiltrating lymphocytes (TILs) were analyzed for *Nr4a3*-Timer, PD1, Lag3, and *Ifng*-YFP expression (Figures 6A–6D). Both CD4^{+} and CD8^{+} TILs exhibited high expression of *Nr4a3* at both days 11 and 14. High frequencies of PD1^{+} and Lag3^{+} T cells was observed at day 11 and day 14, with CD8^{+} T cells significantly more enriched for PD1^{+} and Lag3+ T cells (Figure 6C). *Ifng*-YFP production was found within the CD8^{+} TILs, and a significant proportion were *Nr4a3*-Blue^{+}—indicating active TCR signaling (Figure 6D). This confirmed that the MC38 model exhibits hallmarks of both CD4^{+} and CD8^{+} T cell responses and would serve as a useful model for investigating T cell signatures

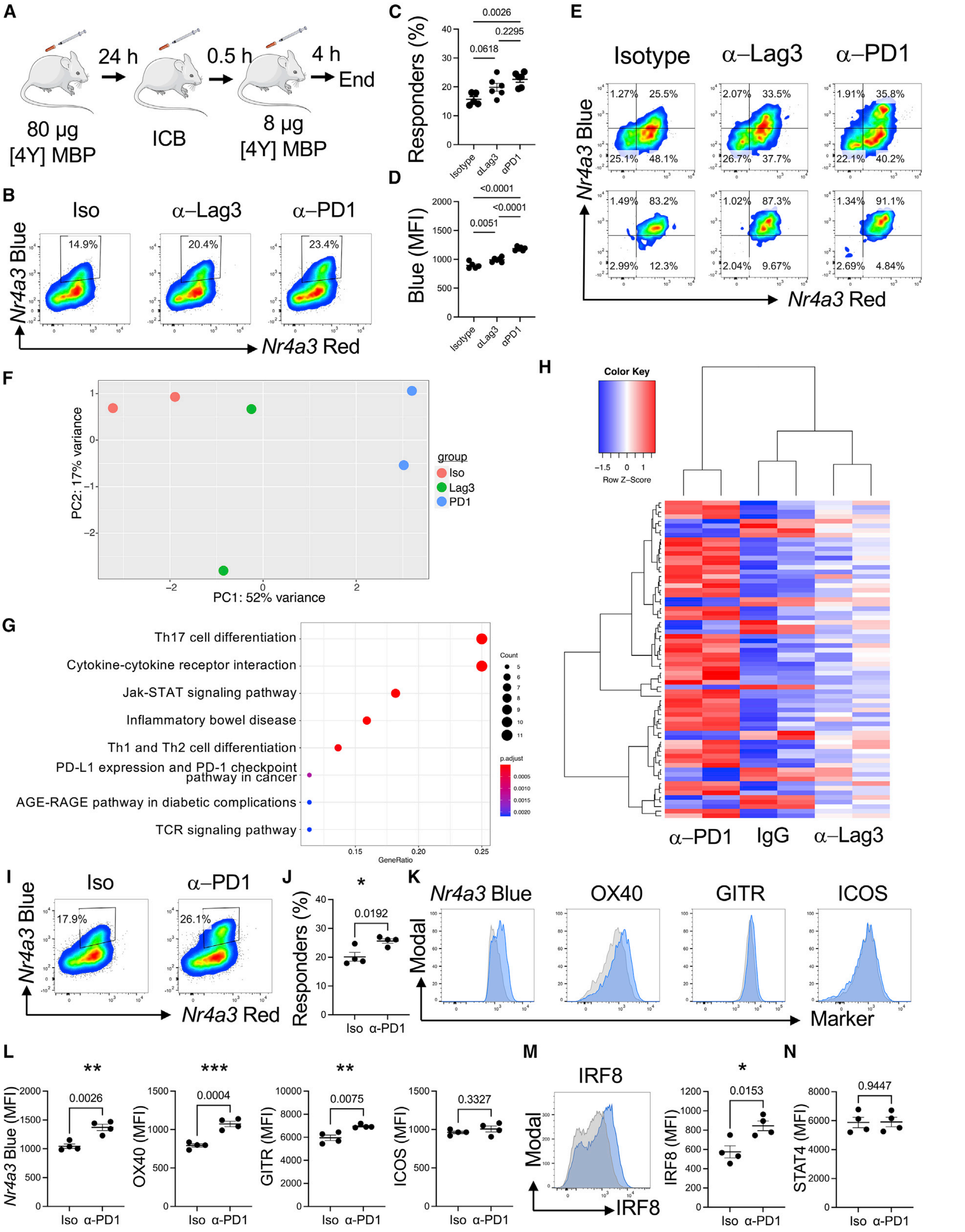

(legend on next page)

CellPress
OPEN ACCESS

of response to immune checkpoint blockade. We analyzed a previously published RNA-seq dataset performed in mice transplanted with MC38 tumors that were subsequently treated with anti-PD-L1 or isotype control (Efremova et al., 2018). We identified 357 genes that were upregulated, and heatmap analysis showed strong signatures in 2 out of 3 anti-PD-L1-treated mice, with an intermediate signature in the third anti-PD-L1 treated mouse (Figure 6E). We interrogated the 28 out of 51 genes identified from our analyses in Figure S5A (upregulated in both 80 μg versus 0.8 μg and anti-PD1 versus isotype datasets) to visualize expression of these genes in the Efremova et al. (2018) dataset (Figure 6F; 25 out of the 28 genes were detectable in the sequencing data). These data demonstrated that most signature T cell genes identified in Figure 5 were upregulated within the anti-PD-L1 treatment group in this model, including *Tnfrsf4*, *Icos*, *Irf8*, *Chmp4b*, and *Irf4*. This suggests that our T cell signatures identified through the Tg4 *Nr4a3*-Tocky model are faithful at discriminating T cell responses in an anti-PD-L1-responsive tumor model.

Strong TCR signaling is a hallmark of melanoma-patient responders to anti-PD1 immunotherapy

Our findings that anti-PD1 imparts signatures of strong TCR signaling led us to hypothesize that genes upregulated in response to strong TCR signaling (Figure 2) or genes upregulated in T cells reactivated in presence of anti-PD1 (Figure 5) could be useful for identifying T cell-intrinsic correlates of response to PD1 immunotherapy. We analyzed a human gene expression dataset of biopsies from advanced melanoma patients before and after nivolumab (anti-PD1) therapy. DEGs were examined in those on therapy (OT; DEGs between pre- and on-therapy samples, regardless of response) or in those with evidence of clinical response (Res; DEGs between pre- and on-therapy samples, considering genes that change differentially in responders versus non-responders) (Riaz et al., 2017). We intersected these genes with DEGs from our Tg4 *Nr4a3*-Tocky datasets: 4-h T cells stimulated with 80 μg versus 0.8 μg (4 h) and DEGs in T cells re-activated for 4 h in the presence of anti-PD1 versus isotype (PD1). Almost all genes in the OT group were also found within the DEGs in those who exhibited signs of clinical response (Figure 7A; Table S4). We identified 6 gene groups intersecting between responders and 4-h strong TCR stimulation or anti-PD1 (or both). Intersection of 4-h and anti-PD1 datasets showed that 2 genes, *ICOS* and *TNIP3,* were associated with both anti-PD1 and strong TCR signaling in mice as well as clinical response to nivolumab, although these genes also changed in patients on therapy regardless of response (group I). This indicates that these may be pharmacodynamic correlates of anti-PD1 therapy. *TNFRSF4* (OX40), *IRF8*, and *STAT4* genes were upregulated only in patients who clinically responded to nivolumab and were predicted from our strong 4-h TCR and murine anti-PD1-specific T cell signatures (Figure 7A, group II). Genes such as *IFNG*, *GZMB* (T cell effector cytokines), and *CTLA4* (checkpoint) were upregulated in the 4-h strong TCR signaling group but also in both the clinical responders and on-therapy groups, suggesting that these also show pharmacodynamic responses (group III). Further analysis between clinical response and strong TCR signaling in murine T cells showed that genes associated with immune activation (*IL2RA*) were upregulated in T cells exhibiting strong TCR signals and only in those patients benefiting from nivolumab (group IV). Furthermore, we identified *CD5*, *GPR65*, and *GCNT1* as a motif that is upregulated on T cells in response to anti-PD1 blockade in mice as well as only in melanoma patients who respond to nivolumab (group V) and *TNFRSF9* (CD137, T cell activation marker) as upregulated in responder and on-therapy groups as well as in anti-PD1-treated Tg4 *Nr4a3*-Tocky T cells. Based on these findings, we selected genes in group I (*ICOS*, *TNIP3*) and group II

Figure 5. Co-inhibitory receptors exert distinct quantitative and qualitative control over T cell re-activation

(A) Experimental design for blockade of co-inhibitory receptors.

(B) Tg4 *Nr4a3*-Tocky *Il10*-GFP mice were immunized s.c. with 80 μg of [4Y] MBP. 24 h later mice were randomized to receive either 0.5 mg isotype pool (1:1 ratio of rat IgG1 and rat IgG2a), anti-Lag3, or anti-PD1 30 min prior to re-challenge with 8 μg [4Y] MBP peptide. Splenic $CD4^+$ T cells were analyzed for *Nr4a3*-Blue versus *Nr4a3*-Red analysis 4 h after peptide re-challenge.

(C and D) Summary data from (B) detailing the percentage of responders (percent of *Nr4a3*-$Blue^+Red^{+,}$ C) or median *Nr4a3*-Blue within *Nr4a3*-$Blue^+Red^+$ $CD4^+$ T cells (D) in isotype (n = 5), anti-Lag3 (n = 6), or anti-PD1 (n = 6) treated mice. Bars represent mean ± SEM, dots represent individual mice. Statistical analysis by one-way ANOVA with Tukey's multiple comparisons test.

(E) Tg4 *Nr4a3*-Tocky *Il10*-GFP mice were immunized s.c. with 80 μg of [4Y] MBP. 24 h later, mice were randomized to receive 0.8 mg isotype pool, 0.8 mg anti-Lag3, or 0.8 mg anti-PD1 30 min prior to re-challenge with 8 μg [4Y] MBP peptide. Splenic $CD4^+$ T cells expression of *Nr4a3*-Blue versus *Nr4a3*-Red 4 h after peptide re-challenge in pre-sorted (top) and sorted (bottom) populations.

(F) RNA was extracted from the sorted populations and 3′ mRNA-seq performed. PCA of the normalized expression data identified 3 clusters, n = 2.

(G) KEGG pathway analysis of DEG between isotype and anti-PD1 treated groups.

(H) Z score heatmap analysis of log2 transformed and normalized counts displaying the 69 DEG between isotype and anti-PD1 groups, in isotype, anti-PD1, or anti-Lag3 groups.

(I) Tg4 *Nr4a3*-Tocky *Il10*-GFP mice were immunized s.c. with 80 μg of [4Y] MBP. 24 h later mice received 0.5 mg rat IgG2a or or anti-PD1 30 min prior to re-challenge with 8 μg [4Y] MBP peptide. Splenic $CD4^+$ T cells were analyzed for *Nr4a3*-Blue versus *Nr4a3*-Red analysis 4 h after peptide re-challenge. Gates were set on responding T cells.

(J) Summary data from (I) for cells responding to dose 2 (n = 4). Bars represent mean ± SEM. *p < 0.05 by unpaired t test.

(K) Histograms showing expression of *Nr4a3*, OX40, GITR and ICOS in responding T cells (indicated by gates in I) between isotype (gray)- or anti-PD1 (blue)-treated cells.

(L) Summary data of the median of expression of the stated markers in responding T cells, n = 4. Bars represent mean ± SEM. Statistical analysis by unpaired t test. **p < 0.01, ***p < 0.001.

(M) Analysis of intracellular IRF8 in $CD4^+$ Tg4 T cells from isotype (gray)- or anti-PD1 (blue)-treated mice, n = 4. Bars represent mean ± SEM. Statistical analysis by unpaired t test. *p < 0.05..

(N) Analysis of intracellular STAT4 in isotype or anti-PD1 treated mice. Bars represent mean ± SEM, n = 4.

Please also see Figures S4 and S5 and Table S3.

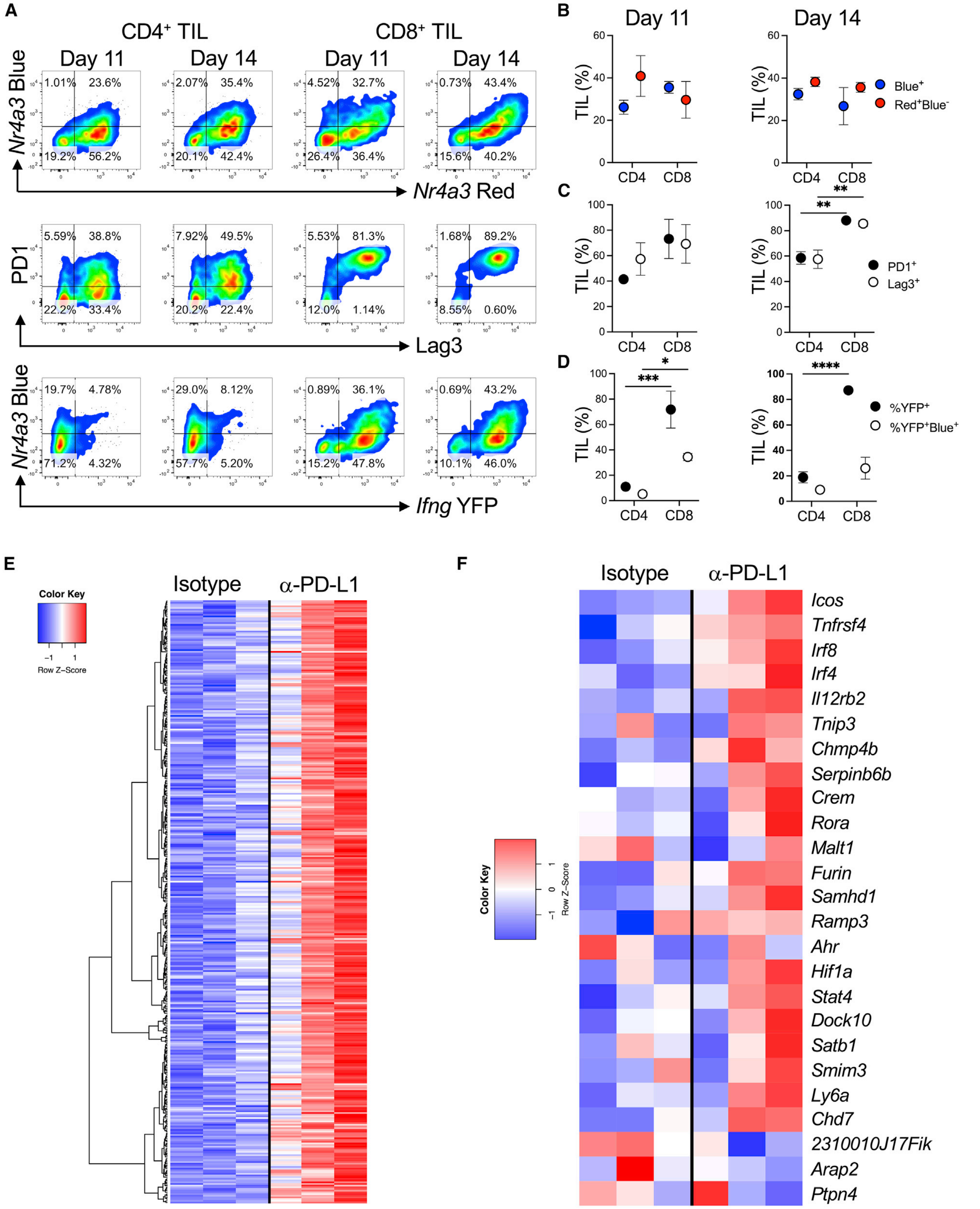

Figure 6. Strong TCR signaling signatures in tumors of anti-PD-L1-treated mice

(A) 0.25 M MC38 cells were injected s.c. into *Nr4a3*-Tocky *Ifng*-YFP mice. CD4$^+$ and CD8$^+$ TILs were analyzed for *Nr4a3*-Blue versus Red (top), PD1 versus Lag3 (middle), or *Nr4a3*-Blue versus *Ifng*-YFP (bottom) expression.

(legend continued on next page)

(*TNFRSF4*, *IRF8*, *STAT4*) as the basis for creating a transcriptional signature metric for strong TCR signaling (TCR.strong). We reckoned that by combining indicators of T cell pharmacodynamic responses to anti-PD1 (*ICOS*, *TNIP3*), with genes changing in patients showing clinical benefit that are also associated with strong TCR signaling and anti-PD1-specific T cell changes (*TNFRSF4*, *IRF8*, *STAT4*), we could develop a useful metric to stratify patient responses to therapy. We utilized an analogous approach to that taken for the cytolytic score metric, where the geometric mean is taken for the transcripts per million (TPM) from sequencing data (Rooney et al., 2015). For comparison, we selected a canonical T cell activation gene set, which incorporated *IL2RA, NR4A1*, *CD69*, and *TNFRSF9* (Figure S7A). The TCR.strong metric was enriched in the MC38 RNA-seq pre-clinical model, but no clear change was seen in the T activation score (Figure S7B). Using our TCR.strong metric, we interrogated the Riaz et al. (2017) dataset for nivolumab patients based on their responses and ipilimumab status (Table S5). Pre-therapy biopsies displayed no difference in TCR.strong or T cell activation metrics between responder (R) and non-responder (NR) groups (Figure S7C). Analyzing the on-therapy cohort revealed that TCR.strong was enriched in R compared with NRs but that the differences in the T activation score was not statistically significant (Figure 7B). The Riaz et al. cohort contains a mix of patients who had previously been on anti-CTLA-4 therapy (ipilimumab, Ipi) and had progressed onto anti-PD1 (Ipi-P) or who were previously Ipi-Naive (Ipi-N, i.e., no previous immunotherapy). Analysis of the TCR.strong metric in the Ipi-N cohort of patients showed increases in those with clinical responses ($p = 0.0105$) and a strong trend in patients from the Ipi-P group ($p = 0.1531$, Figure 7C). No significant differences were found in the same patient groups using the T activation metric. To determine the change in the TCR.strong metric before and after therapy, we identified all patients with pre- and on-therapy biopsies who had known clinical outcomes (Figures 7D and 7E; Table S5). In both the Ipi-P and Ipi-N cohorts' NRs had no change in TCR.strong metric, suggesting that the TCR.strong metric is not influenced by anti-PD1 pharmacodynamics. For the Ipi-N cohort, this increase in TCR.strong score was highly significant ($p = 0.00097$) in those with evidence of clinical benefit, which was not captured by the T cell activation metric (Figure 7D). However, Ipi-P patients with clinical responses displayed a significant increase in both their TCR.strong and T cell activation metrics (Figure 7E) from pre-therapy amounts. By splitting the cohort by median TCR.strong or T cell activation values, TCR.strong "High" patients showed a significant increase in progression free survival (PFS) across the whole cohort compared to "Low" patients which was not the case with T cell activation (Figure 7F). Analysis of overall survival (OS) in the Ipi-N and Ipi-P groups identified significantly increased survival in the TCR.strong "High" group in Ipi-N patients but not in any cohort using the T cell activation "High" group (Figure 7G). To visualize TCR.strong differences at the gene level, heatmap analysis showed a more consistent pattern in the Ipi-N group (Figure 7H) compared with Ipi-P patients (Figure 7I).

We validated the metric through utilizing early-during-treatment (EDT) patients from the (Gide et al., 2019) melanoma cohort. TCR.strong metric showed a higher degree of statistical significance for separating R compared with NR patient groups compared with the T cell activation metric (Figure 7J; TCR.strong, $p = 0.0012$; T cell activation metric $p = 0.0441$; Table S6). The cohort reported in Gide et al. is a combination of EDT patients biopsied within the first two weeks of either commencing anti-PD1 monotherapy or PD1 and CTLA4 combination therapy. Splitting the patient cohort by therapy status showed that both patient subgroups had very strong trends for increased TCR.strong scores in R versus NRs (Figure 7K). Analysis of the combined cohort revealed that patients with a "High" TCR.strong score had significantly increased PFS ($p = 0.0034$) and OS ($p = 0.017$) compared with the "Low" patients (Figure 7L). In summary, our findings demonstrated how analysis of TCR signal strength can inform the outcomes for patients on anti-PD1 immunotherapy.

DISCUSSION

In this study, we demonstrated how antigen abundance and immune checkpoints modulate the strength of TCR signaling and the early T cell activation process. As the frequency of a given TCR precursor in a polyclonal setting influences its magnitude of response to an antigen (Moon et al., 2007), we employed a TCR transgenic approach to focus solely on T cells within the same clonal niche. Through manipulating T cell responses to a modified self-antigen, we identified basic immunological mechanisms that drive the recalibration of T cell activation thresholds and refine a TCR signal strength metric that can monitor melanoma patient responses to nivolumab.

The Tg4 TCR transgenic model allowed us to make robust analyses of systemic T cell responses, as our model leads to the rapid and synchronized activation of T cells. Hence, we were able to follow the activation trajectories of peripheral T cells experiencing different strengths of TCR signaling. Our findings identified several facets of T cell activation that appear to be rapidly programmable because of the strength of TCR signals experienced. We have provided evidence that strong TCR signaling leads to the early upregulation of multiple Th pathways within the same clonal niche, with a bias toward pathways associated with Th1, Th17, and Tr1 cells. While these findings echo

(B–D) Summary data of percentage of TIL for (B) *Nr4a3*-Blue$^+$ (blue) or *Nr4a3*-Red$^+$Blue$^-$ (red), (C) PD1$^+$ (black) or Lag3$^+$ (white), and (D) *Ifng*-YFP$^+$ (black) or percentage of *Ifng*$^+$*Nr4a3*-Blue$^+$ (white), n = 3. Circles represent mean ± SEM. Statistical analysis by two-way Anova with Sidak's multiple comparisons test. *$p < 0.05$, **$p < 0.01$, ***$p < 0.001$, ****$p < 0.0001$.

(E) Heatmap of log2 transformed and normalized counts for genes significantly upregulated (>1.5-fold and adjusted p value < 0.05) in C57BL/6 mice injected with 0.5 M MC38 cells then treated with isotype or anti-PD-L1 every 3 to 4 days before whole tumors were excised and 3′ mRNA-seq was performed (GEO: GSE93018) (Efremova et al., 2018).

(F) Z score heatmap analysis of log2 transformed and normalized counts for genes pre-selected from Figure S5A and also expressed in GEO: GSE93018 (Efremova et al., 2018).

See also Figure S6.

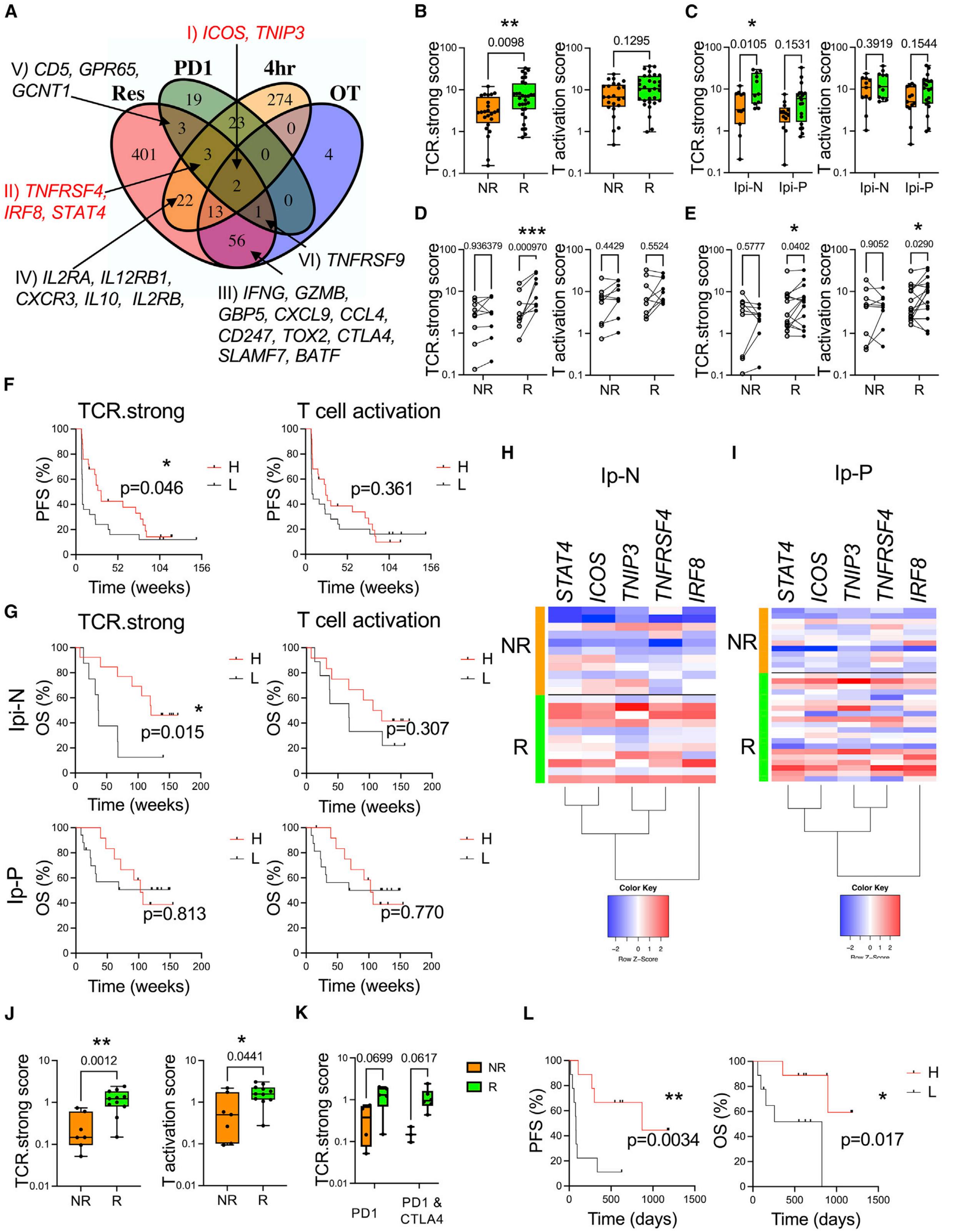

Figure 7. Identification of a strong TCR signal metric that stratifies melanoma patient responses to nivolumab therapy

(A) DEG from 4-h time point of 80 μg versus 0.8 μg [4Y] MBP (4 h; Figure 2), isotype versus anti-PD1 (PD1; Figure 5) were intersected with DEG from melanoma patients who received nivolumab therapy (Riaz et al., 2017). The DEG in these patients were then classified based on (1) the change in expression that occurred on

(legend continued on next page)

CellPress
OPEN ACCESS

the concept that Th1 cell development depends on the strength of TCR signals compared with Th2 cells (Constant et al., 1995), other studies have identified that TCR signal strength plays a key role in directing CD4$^+$ T cell differentiation (Tubo and Jenkins, 2014). Our data, however, suggest that great heterogeneity exists in the early T cell response that does not fit to a simplified model of Th cell differentiation. These findings echo recent reports of gut CD4$^+$ T cell programs displaying a continuum of phenotypes (Kiner et al., 2021).

Our approach allowed us to compare only T cells that had very recently activated the NFAT-*Nr4a3* pathway *in vivo* (Jennings et al., 2020), allowing us to control for the relative frequency of responders while also comparing them at similar phases following TCR ligation. This approach allowed us to interpret the kinetics of the activation of key T cell modules. *Gzmb* and *Ifng*, which are hallmark genes of CD8$^+$ T cell responses, were primed in both weak and strong TCR signaling conditions but with differing kinetics. These data suggest that, within T cells that cross the NFAT-*Nr4a3* activation threshold, some modules are primed regardless of the TCR signal strength—a finding shown clearly for CD8$^+$ T cells and their cytolytic capacity *in vitro* (Richard et al., 2018). However, our findings also reflect that the speed and duration with which these modules are activated differ, with evidence of sustained activation in T cells experiencing a strong TCR signal. In addition, signatures of strong TCR signaling were evident—including the Th17-associated program, enzymes involved in zinc bioavailability (*Mt1*, *Mt2*, linked to T cell exhaustion; Singer et al., 2016) and sustained activation of *Malt1*. Malt1 has essential roles in NF-κB activation in T cells (Rebeaud et al., 2008), and it is tempting to speculate that its function is important in switching NF-κB to full and binary activation that is not achieved by less potent TCR ligands (Gallagher et al., 2020). In addition, it is worth noting that *Tnip3* (a TCR.strong gene) also has roles in regulating the NF-κB pathway.

It has been proposed previously that self-peptide and MHC abundance may tune the responses of T cells to antigen (Grossman and Paul, 2015), as shown that those with higher expression of Nur77 and CD5 exhibit hallmarks of T cell anergy (such as PD1 and Cbl expression; Zinzow-Kramer et al., 2019). Our approach revealed how TCR signal strength primes key immune checkpoints with relevance to immunotherapy. Notably CTLA-4 was heavily influenced by the strength of TCR signaling, giving a graded response to antigen dose *in vivo*. In contrast, PD1 showed a modest reduction in response to weaker TCR signals, highlighting that PD1 is tightly linked to the activation process. Lag3, Tigit, and *Il10* appeared as a delayed and transient module, which was a feature of the stronger TCR signaling group. Similar observations have been observed in chronic tolerance models (Burton et al., 2014) and in models of persisting antigen (Trefzer et al., 2021).

Our findings revealed that very strong TCR signaling leads to a rapid recalibration of T cell activation thresholds at 24 h. We believe this is not because of negative feedback regulation of the TCR signalosome (as CD4 and TCR complexes rapidly recover to control amounts by 24 h) but a re-wired T cell activation state akin to adaptive tolerance (Chiodetti et al., 2006). Furthermore, this state is dynamic since it can be overcome by increasing the TCR signal strength or through the blockade of negative regulators such as PD1 and Lag3. Increased time between immunizations restored sensitivity to lower antigen doses, indicating that single immunization-driven recalibration of activation threshold is reversible at this stage of the immune response. This tunable activation threshold allowed us to directly compare the potencies of PD1 and Lag3 in controlling the early re-activation of T cells *in vivo*. Our data clearly show that both exert quantitative control on the frequencies of re-activating T cells; however, anti-PD1 showed clear qualitative *in vivo* effects, echoing some recent *in vitro* studies (Shimizu et al., 2020). Other recent data have suggested that Lag3 has a complex mechanism of

therapy regardless of response compared with pre-therapy samples (OT) and (2) DEG that changed compared with pre-therapy samples in those patients showing clinical responses (Res). For human datasets, a log fold-change > 0.5 and adjusted p value < 0.1 was set. Genes of interest within the sets are annotated. Full lists of genes upregulated in the four datasets are listed in Table S4.

(B) TCR.strong (left) or T activation scores in on-therapy samples in responder (green, n = 31) and non-responder (orange, n = 24) statistical analysis by Mann-Whitney U test. Box plot with bars displaying median and IQR and whiskers the min and max values. **$p < 0.01$.

(C) TCR.strong or T activation score in on-therapy samples in responder (green, Ipi-P n = 20, Ipi-N n = 11) and non-responder (orange, Ipi-P n = 13, Ipi-N n = 11) patients. Box plot with bars displaying median and IQR and whiskers the min and max values. Statistical analysis by two-way ANOVA with Sidak's multiple compaisons test. *$p < 0.05$.

(D) TCR.strong or T activation scores in Ipi-N patients before and after therapy (paired samples indicated by lines), in non-responder (NR, n = 9) or responder (R, n = 9). Statistical analysis by repeated measures two-way ANOVA with Sidak's multiple compaisons test. ***$p < 0.001$.

(E) TCR.strong or T activation scores in Ipi-P patients before and after therapy in non-responder (NR, n = 9) or responder (R, n = 15). Statistical analysis by repeated measures two-way ANOVA with Sidak's multiple compaisons test. *$p < 0.05$.

Dots represent individual patients and lines pairing of samples in (D) and (E). Statistical analysis by two-way ANOVA with Sidak's multiple comparisons test.

(F) Kaplan Meier progression free survival (PFS) curves based on median TCR.strong or T activation scores (n = 50). Statistical analysis by Log-rank test, *$p < 0.05$.

(G) Kaplan Meier survival curves for melanoma patients in Ipi-N (top, n = 21) or Ipi-P (bottom, n = 29) based on median (from F) TCR.strong (left) or T activation (right scores). Statistical analysis by Log-rank test, *$p < 0.05$.

(H and I) Comparison of survival curves by log-rank test. Z score heatmap analysis of log2 transformed and normalized counts for TCR.strong metric genes in Ipi-N (H) or Ipi-P (I) patients. Orange indicates non-responder patients, green indicates responder.

(J) TCR.strong (left) or T activation scores (right) in early during therapy samples from (Gide et al., 2019) in responder (green, n = 11) and non-responder (orange, n = 7), Box plot with bars displaying median and IQR and whiskers the min and max values. statistical analysis by Mann-Whitney U test. *$p < 0.05$, **$p < 0.01$.

(K) TCR.strong scores in responder (green, anti-PD1 n = 5; anti-PD1 and anti-CTLA4 n = 6) and non-responder (orange, anti-PD1 n = 4, anti-PD1 and anti-CTLA4 n = 3) patients. Box plot with bars displaying median and IQR and whiskers the min and max values. Statistical analysis by two-way ANOVA with Sidak's multiple comparisons test.

(L) Kaplan Meier PFS (left) or OS (right) curves split by median TCR.strong scores (n = 18) in (Gide et al., 2019) early during therapy cohort. Statistical analysis by Log-rank test, *$p < 0.05$, **$p < 0.01$.

Please also see Figure S7 and Tables S4, S5, and S6.

action, but it is likely that Lag3 at least in part functions through administering inhibitory signals (Maruhashi et al., 2018). Our data support a weak role in controlling NFAT-*Nr4a3* pathway activation, with no evidence that it substantially alters the quality of the resulting TCR signal. This contrasts with PD1, which imparted features of strong TCR signaling on re-activating T cells. The signature of T cells re-activated in the presence of anti-PD1 re-capitulated many pathways seen in earlier analyses comparing weak and strong TCR signaling. Once again, a bias toward Th1 and Th17 cell-type pathways was evident. In fact, 28 out of 51 genes identified in these analyses overlapped with genes upregulated by strong TCR signals. This finding suggested that *in vivo*, anti-PD1 can directly target TCR signal strength, as has been faithfully shown *in vitro* (Latchman et al., 2001; Mizuno et al., 2019). Anti-PD1 has been proposed to target the anti-CD28 co-stimulatory pathway (Hui et al., 2017), but here in our study, CD28 agonism had no effect on T cell re-activation, suggesting that our data support a key role for anti-PD1 to modify the TCR-driven NFAT-*Nr4a3* pathway *in vivo*.

Given the transcriptional features of T cells reactivating in the presence of anti-PD1, we interrogated to what extent strong TCR signatures are evident in human datasets of melanoma patients undergoing PD1 pathway therapy (Gide et al., 2019; Riaz et al., 2017). It has been clearly documented that IFN-γ signatures are a key part of the anti-PD1 response (Grasso et al., 2020; Riaz et al., 2017). In addition, many T cell signatures have been reported to be predictive for anti-PD1 response in a variety of tumor types, including cytolytic (Rooney et al., 2015), T cell IFN-γ-related mRNA profiles (Ayers et al., 2017), the chemokine *CXCL9* (Chow et al., 2019; House et al., 2020; Litchfield et al., 2021), *CD8A* (Tumeh et al., 2014), and an antagonistic inflammatory phenotype (Bonavita et al., 2020). A large recent meta-analysis concluded that a compound signature involving tumor mutational burden, *CXCL9*, UV, APOBEC, and tobacco signatures can identify pan-cancer responses to ICB (Litchfield et al., 2021). Identification of biomarkers of ICB efficacy before treatment commences would be ideal, as patients could be given treatment based on the likelihood that they will respond. However, given that most patients do not respond to ICB (Borcoman et al., 2019; Sharma et al., 2017), such a test would need to ethically have a very high positive predictive value for widespread clinical application to avoid the potential denial of patients for life-extending treatments. Our analysis shows that hallmark signatures of strong TCR signaling can stratify the outcomes of patients on anti-PD1 pathway therapy. Our TCR.strong metric comprised of 5 immunological genes (*TNFRSF4*, *IRF8*, *STAT4*, *TNIP3*, *ICOS*—the latter previously identified as a potential marker for T cell mediated response to anti-PD1 monotherapy in melanoma; Xiao et al., 2020). TCR.strong genes were upregulated rapidly in T cells (<4 h) either experiencing a primary strong TCR signal or in T cells re-activated in the presence of anti-PD1. The TCR.strong metric was not altered in patients without clinical response, suggesting that these genes are less sensitive to potential pharmacodynamic effects of anti-PD1 therapy. It also demonstrates that this metric cannot predict patient responses before the onset of therapy. However, there remains an urgent need to identify markers to monitor treatment efficacy in patients, to inform clinical decision making, and to enhance the implementation of precision immunotherapy (Havel et al., 2019). In addition, we anticipate that as increasing numbers of ICB combinations become available, identifying signatures for treatment monitoring for efficaciousness will become increasingly as important as identifying predictive biomarkers.

In summary, our study provides insight into how TCR signal strength and its manipulation control the T cell activation process. Co-inhibitory receptors rapidly re-calibrate the activation threshold of T cells, and we demonstrate how anti-PD1 leads to a strong TCR signal strength signature that is a correlate for survival of melanoma patients on anti-PD1 monotherapy.

Limitations of study

The central model utilized here employs a tolerogenic immunization, which likely does not fully capture all aspects of the tumor environment, where co-stimulation has been shown to play an important role (Kamphorst et al., 2017). In addition, our analyses are limited to early recalibration events (first 24–48 h) following TCR signals, which means that the extent to which these findings relate to the later stages of immune responses (e.g., sequential epigenetic changes that may occur in exhausted T cells) are unclear. The TCR.strong metric has been predicted using a $CD4^+$ T cell system and then applied at the bulk tumor level, therefore the extent to which this metric is modified in $CD8^+$ versus $CD4^+$ T cells (or potentially other cells) remains to be determined.

STAR★METHODS

Detailed methods are provided in the online version of this paper and include the following:

- KEY RESOURCES TABLE
- RESOURCE AVAILABILITY
  - Lead contact
  - Materials availability
  - Data and code availability
- EXPERIMENTAL MODEL AND SUBJECT DETAILS
  - Mice
- METHOD DETAILS
  - *In vitro* cultures
  - Immunisations
  - Antibody treatments
  - Flow cytometry and cell sorting
  - MC38 model
  - RNA-seq library preparation and analysis
  - Analysis of published human anti-PD1 and MC38 anti-PD-L1 datasets
  - Generation and implementation of TCR.strong metric
- QUANTIFICATION AND STATISTICAL ANALYSIS

SUPPLEMENTAL INFORMATION

Supplemental information can be found online at https://doi.org/10.1016/j.immuni.2021.08.020.

ACKNOWLEDGMENTS

Work funded by the University of Birmingham (D.B.), the Wellcome Trust (214018/Z/18/Z to D.B.), and the MRC (MR/V009052/1 to D.B.). D.A.L. is funded by a Wellcome Trust 4-year Basic Science PhD program. E.K.J. is supported by a studentship from the MRC Discovery Medicine North (DiMeN)

CellPress
OPEN ACCESS

Doctoral Training Partnership (MR/N103840/1). A.C. and K.M.M. are funded by a CRUK Career Establishment Award (C61638/A27112 to K.M.M.). D.C.W and A.F-L. are funded by the University of Birmingham (UoB). Diagrams in Figures 1, 4, and 5 were adapted from the Servier Medical Art templates, which are licensed under a CC BY 3.0 unported license, https://smart.servier.com. We thank Dr. Leila Khoja, Dr. Neil Steven, and Dr. Lalit Pallan (Medical Oncology, UoB) for helpful discussion around the potential clinical utility of the TCR.strong metric. We also thank Dr. Sarah Dimeloe, Dr. Rebecca Drummond, and Dr. Wei-Yu Lu for their continued support and feedback on data in the manuscript. The graphical abstract was created using BioRender.com.

AUTHOR CONTRIBUTIONS

Conceptualization, funding acquisition, supervision, formal analysis, methodology, data curation, project administration, and writing of original draft, D.B. T.A.E.E., E.K.J., N.T., D.A.J.L., A.C., and D.B. performed and analyzed experiments. D.B. performed and analyzed RNA-seq experiments and performed all bioinformatic analyses, including conceptualization (together with T.A.E.E.) and implementation of the TCR.strong metric. D.C.W. provided resources and advice on methodology for Tg4 model immunization. A.F.-L performed cell sorting experiments. K.M.M. funding acquisition. T.A.E.E. and D.B. wrote the paper and all authors were involved in reviewing the original draft manuscript.

DECLARATION OF INTERESTS

The authors declare no competing interests.

INCLUSION AND DIVERSITY

We worked to incorporate sex balance in the selection of non-human subjects. One or more of the authors of this paper self-identifies as a member of the LGBTQ+ community.

Received: March 4, 2021
Revised: June 21, 2021
Accepted: August 17, 2021
Published: September 16, 2021

REFERENCES

Altan-Bonnet, G., and Germain, R.N. (2005). Modeling T cell antigen discrimination based on feedback control of digital ERK responses. PLoS Biol. *3*, e356.

Ayers, M., Lunceford, J., Nebozhyn, M., Murphy, E., Loboda, A., Kaufman, D.R., Albright, A., Cheng, J.D., Kang, S.P., Shankaran, V., et al. (2017). IFN-γ-related mRNA profile predicts clinical response to PD-1 blockade. J. Clin. Invest. *127*, 2930–2940.

Bending, D., Paduraru, A., Ducker, C.B., Prieto Martín, P., Crompton, T., and Ono, M. (2018a). A temporally dynamic *Foxp3* autoregulatory transcriptional circuit controls the effector Treg programme. EMBO J. *37*, e99013.

Bending, D., Prieto Martín, P., Paduraru, A., Ducker, C., Marzaganov, E., Laviron, M., Kitano, S., Miyachi, H., Crompton, T., and Ono, M. (2018b). A timer for analyzing temporally dynamic changes in transcription during differentiation in vivo. J. Cell Biol. *217*, 2931–2950.

Bevington, S.L., Ng, S.T.H., Britton, G.J., Keane, P., Wraith, D.C., and Cockerill, P.N. (2020). Chromatin Priming Renders T Cell Tolerance-Associated Genes Sensitive to Activation below the Signaling Threshold for Immune Response Genes. Cell Rep. *31*, 107748.

Bonavita, E., Bromley, C.P., Jonsson, G., Pelly, V.S., Sahoo, S., Walwyn-Brown, K., Mensurado, S., Moeini, A., Flanagan, E., Bell, C.R., et al. (2020). Antagonistic Inflammatory Phenotypes Dictate Tumor Fate and Response to Immune Checkpoint Blockade. Immunity *53*, 1215–1229.

Borcoman, E., Kanjanapan, Y., Champiat, S., Kato, S., Servois, V., Kurzrock, R., Goel, S., Bedard, P., and Le Tourneau, C. (2019). Novel patterns of response under immunotherapy. Ann. Oncol. *30*, 385–396.

Borst, J., Ahrends, T., Bąbała, N., Melief, C.J.M., and Kastenmüller, W. (2018). CD4+ T cell help in cancer immunology and immunotherapy. Nat. Rev. Immunol. *18*, 635–647.

Burton, B.R., Britton, G.J., Fang, H., Verhagen, J., Smithers, B., Sabatos-Peyton, C.A., Carney, L.J., Gough, J., Strobel, S., and Wraith, D.C. (2014). Sequential transcriptional changes dictate safe and effective antigen-specific immunotherapy. Nat. Commun. *5*, 4741.

Cachot, A., Bilous, M., Liu, Y.C., Li, X., Saillard, M., Cenerenti, M., Rockinger, G.A., Wyss, T., Guillaume, P., Schmidt, J., et al. (2021). Tumor-specific cytolytic CD4 T cells mediate immunity against human cancer. Sci. Adv. *7*, eabe3348.

Chen, H., and Boutros, P.C. (2011). VennDiagram: a package for the generation of highly-customizable Venn and Euler diagrams in R. BMC Bioinformatics *12*, 35.

Chiodetti, L., Choi, S., Barber, D.L., and Schwartz, R.H. (2006). Adaptive tolerance and clonal anergy are distinct biochemical states. J. Immunol. *176*, 2279–2291.

Chow, M.T., Ozga, A.J., Servis, R.L., Frederick, D.T., Lo, J.A., Fisher, D.E., Freeman, G.J., Boland, G.M., and Luster, A.D. (2019). Intratumoral Activity of the CXCR3 Chemokine System Is Required for the Efficacy of Anti-PD-1 Therapy. Immunity *50*, 1498–1512.

Conley, J.M., Gallagher, M.P., Rao, A., and Berg, L.J. (2020). Activation of the Tec Kinase ITK Controls Graded IRF4 Expression in Response to Variations in TCR Signal Strength. J. Immunol. *205*, 335–345.

Constant, S., Pfeiffer, C., Woodard, A., Pasqualini, T., and Bottomly, K. (1995). Extent of T cell receptor ligation can determine the functional differentiation of naive CD4+ T cells. J. Exp. Med. *182*, 1591–1596.

Das, J., Ho, M., Zikherman, J., Govern, C., Yang, M., Weiss, A., Chakraborty, A.K., and Roose, J.P. (2009). Digital signaling and hysteresis characterize ras activation in lymphoid cells. Cell *136*, 337–351.

Durinck, S., Spellman, P.T., Birney, E., and Huber, W. (2009). Mapping identifiers for the integration of genomic datasets with the R/Bioconductor package biomaRt. Nat. Protoc. *4*, 1184–1191.

Efremova, M., Rieder, D., Klepsch, V., Charoentong, P., Finotello, F., Hackl, H., Hermann-Kleiter, N., Löwer, M., Baier, G., Krogsdam, A., and Trajanoski, Z. (2018). Targeting immune checkpoints potentiates immunoediting and changes the dynamics of tumor evolution. Nat. Commun. *9*, 32.

Gallagher, M.P., Conley, J.M., and Berg, L.J. (2018). Peptide Antigen Concentration Modulates Digital NFAT1 Activation in Primary Mouse Naive CD8+ T Cells as Measured by Flow Cytometry of Isolated Cell Nuclei. Immunohorizons *2*, 208–215.

Gallagher, M.P., Conley, J.M., Vangala, P., Reboldi, A., Garber, M., and Berg, L.J. (2020). The Tec kinase ITK differentially optimizes NFAT, NF-kB, and MAPK signaling during early T cell activation to regulate graded gene induction. bioRxiv. https://doi.org/10.1101/2020.11.12.380725.

Gide, T.N., Quek, C., Menzies, A.M., Tasker, A.T., Shang, P., Holst, J., Madore, J., Lim, S.Y., Velickovic, R., Wongchenko, M., et al. (2019). Distinct Immune Cell Populations Define Response to Anti-PD-1 Monotherapy and Anti-PD-1/Anti-CTLA-4 Combined Therapy. Cancer Cell *35*, 238–255.

Grasso, C.S., Tsoi, J., Onyshchenko, M., Abril-Rodriguez, G., Ross-Macdonald, P., Wind-Rotolo, M., Champhekar, A., Medina, E., Torrejon, D.Y., Shin, D.S., et al. (2020). Conserved Interferon-gamma Signaling Drives Clinical Response to Immune Checkpoint Blockade Therapy in Melanoma. Cancer Cell *38*, 500–515.

Grossman, Z., and Paul, W.E. (2015). Dynamic tuning of lymphocytes: physiological basis, mechanisms, and function. Annu. Rev. Immunol. *33*, 677–713.

Havel, J.J., Chowell, D., and Chan, T.A. (2019). The evolving landscape of biomarkers for checkpoint inhibitor immunotherapy. Nat. Rev. Cancer *19*, 133–150.

Hogan, P.G., Chen, L., Nardone, J., and Rao, A. (2003). Transcriptional regulation by calcium, calcineurin, and NFAT. Genes Dev. *17*, 2205–2232.

Homet Moreno, B., Zaretsky, J.M., Garcia-Diaz, A., Tsoi, J., Parisi, G., Robert, L., Meeth, K., Ndoye, A., Bosenberg, M., Weeraratna, A.T., et al. (2016). Response to Programmed Cell Death-1 Blockade in a Murine Melanoma

Syngeneic Model Requires Costimulation, CD4, and CD8 T Cells. Cancer Immunol. Res. *4*, 845–857.

House, I.G., Savas, P., Lai, J., Chen, A.X.Y., Oliver, A.J., Teo, Z.L., Todd, K.L., Henderson, M.A., Giuffrida, L., Petley, E.V., et al. (2020). Macrophage-Derived CXCL9 and CXCL10 Are Required for Antitumor Immune Responses Following Immune Checkpoint Blockade. Clin. Cancer Res. *26*, 487–504.

Hui, E., Cheung, J., Zhu, J., Su, X., Taylor, M.J., Wallweber, H.A., Sasmal, D.K., Huang, J., Kim, J.M., Mellman, I., and Vale, R.D. (2017). T cell costimulatory receptor CD28 is a primary target for PD-1-mediated inhibition. Science *355*, 1428–1433.

Jennings, E., Elliot, T.A.E., Thawait, N., Kanabar, S., Yam-Puc, J.C., Ono, M., Toellner, K.M., Wraith, D.C., Anderson, G., and Bending, D. (2020). Nr4a1 and Nr4a3 Reporter Mice Are Differentially Sensitive to T Cell Receptor Signal Strength and Duration. Cell Rep. *33*, 108328.

Jennings, E.K., Lecky, D.A.J., Ono, M., and Bending, D. (2021). Application of dual *Nr4a1*-GFP *Nr4a3*-Tocky reporter mice to study T cell receptor signaling by flow cytometry. STAR Protoc *2*, 100284.

Kamanaka, M., Kim, S.T., Wan, Y.Y., Sutterwala, F.S., Lara-Tejero, M., Galán, J.E., Harhaj, E., and Flavell, R.A. (2006). Expression of interleukin-10 in intestinal lymphocytes detected by an interleukin-10 reporter knockin tiger mouse. Immunity *25*, 941–952.

Kamphorst, A.O., Wieland, A., Nasti, T., Yang, S., Zhang, R., Barber, D.L., Konieczny, B.T., Daugherty, C.Z., Koenig, L., Yu, K., et al. (2017). Rescue of exhausted CD8 T cells by PD-1-targeted therapies is CD28-dependent. Science *355*, 1423–1427.

Keck, S., Schmaler, M., Ganter, S., Wyss, L., Oberle, S., Huseby, E.S., Zehn, D., and King, C.G. (2014). Antigen affinity and antigen dose exert distinct influences on CD4 T-cell differentiation. Proc. Natl. Acad. Sci. USA *111*, 14852–14857.

Kiner, E., Willie, E., Vijaykumar, B., Chowdhary, K., Schmutz, H., Chandler, J., Schnell, A., Thakore, P.I., LeGros, G., Mostafavi, S., et al.; Immunological Genome Project Consortium (2021). Gut $CD4^+$ T cell phenotypes are a continuum molded by microbes, not by T_H archetypes. Nat. Immunol. *22*, 216–228.

Latchman, Y., Wood, C.R., Chernova, T., Chaudhary, D., Borde, M., Chernova, I., Iwai, Y., Long, A.J., Brown, J.A., Nunes, R., et al. (2001). PD-L2 is a second ligand for PD-1 and inhibits T cell activation. Nat. Immunol. *2*, 261–268.

Litchfield, K., Reading, J.L., Puttick, C., Thakkar, K., Abbosh, C., Bentham, R., Watkins, T.B.K., Rosenthal, R., Biswas, D., Rowan, A., et al. (2021). Meta-analysis of tumor- and T cell-intrinsic mechanisms of sensitization to checkpoint inhibition. Cell *184*, 596–614.

Love, M.I., Huber, W., and Anders, S. (2014). Moderated estimation of fold change and dispersion for RNA-seq data with DESeq2. Genome Biol. *15*, 550.

Martinez, G.J., Pereira, R.M., Äijö, T., Kim, E.Y., Marangoni, F., Pipkin, M.E., Togher, S., Heissmeyer, V., Zhang, Y.C., Crotty, S., et al. (2015). The transcription factor NFAT promotes exhaustion of activated $CD8^+$ T cells. Immunity *42*, 265–278.

Maruhashi, T., Okazaki, I.M., Sugiura, D., Takahashi, S., Maeda, T.K., Shimizu, K., and Okazaki, T. (2018). LAG-3 inhibits the activation of $CD4^+$ T cells that recognize stable pMHCII through its conformation-dependent recognition of pMHCII. Nat. Immunol. *19*, 1415–1426.

Mizuno, R., Sugiura, D., Shimizu, K., Maruhashi, T., Watada, M., Okazaki, I.M., and Okazaki, T. (2019). PD-1 Primarily Targets TCR Signal in the Inhibition of Functional T Cell Activation. Front. Immunol. *10*, 630.

Moon, J.J., Chu, H.H., Pepper, M., McSorley, S.J., Jameson, S.C., Kedl, R.M., and Jenkins, M.K. (2007). Naive CD4(+) T cell frequency varies for different epitopes and predicts repertoire diversity and response magnitude. Immunity *27*, 203–213.

Moran, A.E., Holzapfel, K.L., Xing, Y., Cunningham, N.R., Maltzman, J.S., Punt, J., and Hogquist, K.A. (2011). T cell receptor signal strength in Treg and iNKT cell development demonstrated by a novel fluorescent reporter mouse. J. Exp. Med. *208*, 1279–1289.

Pachter, L. (2011). Models for transcript quantification from RNA-Seq. arXiv, 1104.3889.

Podtschaske, M., Benary, U., Zwinger, S., Höfer, T., Radbruch, A., and Baumgrass, R. (2007). Digital NFATc2 activation per cell transforms graded T cell receptor activation into an all-or-none IL-2 expression. PLoS ONE *2*, e935.

Price, A.E., Reinhardt, R.L., Liang, H.E., and Locksley, R.M. (2012). Marking and quantifying IL-17A-producing cells in vivo. PLoS ONE *7*, e39750.

Rebeaud, F., Hailfinger, S., Posevitz-Fejfar, A., Tapernoux, M., Moser, R., Rueda, D., Gaide, O., Guzzardi, M., Iancu, E.M., Rufer, N., et al. (2008). The proteolytic activity of the paracaspase MALT1 is key in T cell activation. Nat. Immunol. *9*, 272–281.

Riaz, N., Havel, J.J., Makarov, V., Desrichard, A., Urba, W.J., Sims, J.S., Hodi, F.S., Martin-Algarra, S., Mandal, R., Sharfman, W.H., et al. (2017). Tumor and Microenvironment Evolution during Immunotherapy with Nivolumab. Cell *171*, 934–949.

Richard, A.C., Lun, A.T.L., Lau, W.W.Y., Göttgens, B., Marioni, J.C., and Griffiths, G.M. (2018). T cell cytolytic capacity is independent of initial stimulation strength. Nat. Immunol. *19*, 849–858.

Rooney, M.S., Shukla, S.A., Wu, C.J., Getz, G., and Hacohen, N. (2015). Molecular and genetic properties of tumors associated with local immune cytolytic activity. Cell *160*, 48–61.

Sharma, P., Hu-Lieskovan, S., Wargo, J.A., and Ribas, A. (2017). Primary, Adaptive, and Acquired Resistance to Cancer Immunotherapy. Cell *168*, 707–723.

Shimizu, K., Sugiura, D., Okazaki, I.M., Maruhashi, T., Takegami, Y., Cheng, C., Ozaki, S., and Okazaki, T. (2020). PD-1 Imposes Qualitative Control of Cellular Transcriptomes in Response to T Cell Activation. Mol Cell *77*, 937–950.

Singer, M., Wang, C., Cong, L., Marjanovic, N.D., Kowalczyk, M.S., Zhang, H., Nyman, J., Sakuishi, K., Kurtulus, S., Gennert, D., et al. (2016). A Distinct Gene Module for Dysfunction Uncoupled from Activation in Tumor-Infiltrating T Cells. Cell *166*, 1500–1511.

Stephens, M. (2017). False discovery rates: a new deal. Biostatistics *18*, 275–294.

Subach, F.V., Subach, O.M., Gundorov, I.S., Morozova, K.S., Piatkevich, K.D., Cuervo, A.M., and Verkhusha, V.V. (2009). Monomeric fluorescent timers that change color from blue to red report on cellular trafficking. Nat. Chem. Biol. *5*, 118–126.

Trefzer, A., Kadam, P., Wang, S.H., Pennavaria, S., Lober, B., Akçabozan, B., Kranich, J., Brocker, T., Nakano, N., Irmler, M., et al. (2021). Dynamic adoption of anergy by antigen-exhausted $CD4^+$ T cells. Cell Rep. *34*, 108748.

Tubo, N.J., and Jenkins, M.K. (2014). TCR signal quantity and quality in $CD4^+$ T cell differentiation. Trends Immunol. *35*, 591–596.

Tumeh, P.C., Harview, C.L., Yearley, J.H., Shintaku, I.P., Taylor, E.J., Robert, L., Chmielowski, B., Spasic, M., Henry, G., Ciobanu, V., et al. (2014). PD-1 blockade induces responses by inhibiting adaptive immune resistance. Nature *515*, 568–571.

Xiao, Z., Mayer, A.T., Nobashi, T.W., and Gambhir, S.S. (2020). ICOS Is an Indicator of T-cell-Mediated Response to Cancer Immunotherapy. Cancer Res. *80*, 3023–3032.

Yu, G., Wang, L.G., Han, Y., and He, Q.Y. (2012). clusterProfiler: an R package for comparing biological themes among gene clusters. OMICS *16*, 284–287.

Yu, G., Wang, L.G., Yan, G.R., and He, Q.Y. (2015). DOSE: an R/Bioconductor package for disease ontology semantic and enrichment analysis. Bioinformatics *31*, 608–609.

Zinzow-Kramer, W.M., Weiss, A., and Au-Yeung, B.B. (2019). Adaptation by naïve $CD4^+$ T cells to self-antigen-dependent TCR signaling induces functional heterogeneity and tolerance. Proc. Natl. Acad. Sci. USA *116*, 15160–15169.

STAR★METHODS

KEY RESOURCES TABLE

REAGENT or RESOURCE	SOURCE	IDENTIFIER
Antibodies		
Rat anti mouse CD4 BUV737 (clone GK1.5)	BD Biosciences	Cat# 612761; RRID: AB_2870092
Rat anti-mouse CD8a BUV395 (clone 53-6.7)	BD Biosciences	Cat# 563786; RRID: AB_2732919
Mouse anti-mouse TCR Vb8.1,8.2 BUV395 (clone MR5-2)	BD Biosciences	Cat# 744335; RRID: AB_2742163
Rat anti-mouse CD4 AF700 (clone RM4-4)	BioLegend	Cat# 116022; RRID: AB_2715958
Rat anti-mouse PD1 APC (clone 29F.1A12)	BioLegend	Cat# 135210; RRID: AB_2159183
Rat anti-mouse PD1 PE-Cy7 (clone 29F.1A12)	BioLegend	Cat# 135215; RRID: AB_10696422
Rat anti-mouse TCR Vb8.1,8.2 PerCP-eFluor 710 (clone K716-133)	ThermoFisher	Cat# 46-5813-80; RRID: AB_10548034
Mouse anti-mouse Tigit PE-Cy7 (clone 1G9)	BioLegend	Cat# 142107; RRID: AB_2565648
Rat anti-mouse Lag3 APC (clone C97BW)	BioLegend	Cat# 125209; RRID: AB_10639935
Rat anti-mouse Lag3 PE-Cy7 (clone C97BW)	BioLegend	Cat# 125225; RRID: AB_2715763
Armenian hamster anti-mouse CTLA-4 PE (clone UC10-4B9)	BioLegend	Cat# 106305; RRID: AB_313254
Mouse anti mouse/human IRF8 PE (clone V3GYWCH)	Invitrogen	Cat# 12-9852-80; RRID: AB_2572741
Rabbit anti-mouse STAT4 (clone 2H9L5)	Invitrogen	Cat# 700185; RRID: AB_2532296
F(ab')2-Goat anti-Rabbit IgG (H+L) Cross-Adsorbed Secondary Antibody, APC	Invitrogen	Cat# 31984; RRID: AB_429727
Armenian hamster anti-mouse TCRbeta AF700 (clone H57-597)	BioLegend	Cat# 109224; RRID: AB_1027648
Armenian hamster anti-mouse CD69 APC (clone H1.2F3)	BioLegend	Cat# 104514; RRID: AB_492843
Armenian hamster anti-mouse TCRbeta PerCp-Vy5.5(clone H57-597)	Tonbo Biosciences	Cat# 65-5961-U025; RRID: AB_2621911
Rat anti mouse CD25 PerCP-Cy5.5 (clone PC61)	BioLegend	Cat# 102030; RRID: AB_893288
Rat anti mouse/human CD44 AF700 (clone IM7)	BioLegend	Cat# 103026; RRID: AB_493713
Rat anti mouse CD4 BUV395 (clone GK1.5)	BD Biosciences	Cat# 563790; RRID: AB_2738426
Rat anti-mouse OX40 APC (clone OX-86)	BioLegend	Cat# 119413; RRID: AB_2561723
Rat anti-mouse GITR PE-Cy7 (clone DTA-1)	BioLegend	Cat# 126317; RRID: AB_2563385
Armenian hamster anti human/mouse ICOS AF700 (clone C398.4A)	BioLegend	Cat# 313528; RRID: AB_2566126
Rat anti mouse I-A/I-E PE-Cy7 (clone M5/114.15.2)	BioLegend	Cat# 107629; RRID: AB_2290801
Rat anti-mouse PD-L1 APC (clone 10F.9G2)	BioLegend	Cat# 124311; RRID: AB_10612935
Hamster anti-mouse CD28 (clone 37.51) from hybridoma supernatant	Prof. Anne Cooke (University of Cambridge)	Gift from Prof. Anne Cooke (University of Cambridge
GoInVivo Purified anti-mouse Lag3 (clone C97BW)	BioLegend	Cat# 125216; RRID: AB_2566284
GoInVivo Purified anti-mouse Lag3 (clone C97BW)	BioLegend	Cat# 125217; RRID: AB_2566285
GoInVivo Purified anti-mouse PD-1 (clone 29F.1A12)	BioLegend	Cat# 135233; RRID: AB_2616834

(*Continued on next page*)

Continued		
REAGENT or RESOURCE	SOURCE	IDENTIFIER
InVivo Mab rat anti-mouse PD-1 (clone 29F.1A12)	Bio X Cell	Cat# BE0273; RRID: AB_2687796
Rat IgG1 isotype (clone MAC 221)	Prof. Anne Cooke (University of Cambridge)	Gift from Prof. Anne Cooke (University of Cambridge
Rat IgG2a isotype (clone MAC 219)	Prof. Anne Cooke (University of Cambridge)	Gift from Prof. Anne Cooke (University of Cambridge
Chemicals, peptides, and recombinant proteins		
MBP Ac1-9[4K] peptide AcASQKRPSQR	GL Biochem Shanghai	Custom product
MBP Ac1-9[4A] peptide AcASQARPSQR	GL Biochem Shanghai	Custom product
MBP Ac1-9[4Y] peptide AcASQYRPSQR	GL Biochem Shanghai	Custom product
Phosphate buffered saline (Ca^{2+} Mg^{2+} free)	ThermoFisher	Cat# 14190-094
RPMI 1640 with L-Glutamine	ThermoFisher	Cat# 21875-034
DNASE I, GRADE II	Roche	Cat# 10104159001
Collagenase D	Roche	Cat# 11088858001
Fetal Bovine Serum, qualified, heat inactivated, Brazil	ThermoFisher	Cat# 10500064
Critical commercial assays		
PicoPure™ RNA Isolation Kit	ThermoFisher	Cat# KIT0204
QuantSeq 3¢ mRNA-Seq Library Prep Kit (FWD) for Illumina, 24 preps	Lexogen	Cat# 015.24
eFluor-780 fixable viability dye	eBioscience	Cat# 65-0865-14
MoJo Sort nanobeads: naive CD4 T Cell Isolation Kit	BioLegend	Cat# 480039
MoJo Sort nanobeads: CD90.2 selection Kit	BioLegend	Cat# 480101
eBioscience™ Foxp3 / Transcription Factor Staining Buffer Set	ThermoFisher	Cat# 00-5523-00
eBiosceince 1X RBC lysis buffer	ThermoFisher	Cat# 00-4333-57
Deposited data		
Raw and processed sequencing data for TCR signal strength analysis in Figure 2	This paper	GEO: GSE165817
Raw and processed sequencing data for effect of anti-PD1 in Figure 5	This paper	GEO: GSE165818
Nivolumab pre and on therapy RNaseq processed FPKM and rLog data from Riaz et al. cohort	Riaz et al., 2017	GEO: GSE91061
Gide early during treatment with anti-PD1 melanoma cohort, raw sequencing data	Gide et al., 2019	ENA: PRJEB23709
Riaz et al., patient clinical outcome data	https://github.com/riazn/bms038_analysis/tree/master/data	https://github.com/riazn/bms038_analysis/tree/master/data
Gide et al., patient clinical outcome data	Gide et al., 2019	PMID: 30753825
MC38 colorectal cell line response to anti-PD-L1 sequencing data	Efremova et al., 2018	GEO: GSE93018
Experimental models: Cell lines		
Cancer cell line: MC38	Prof. David Withers (University of Birmingham)	CVCL_B288
Experimental models: Organisms/strains		
Mouse: *Nr4a3*-Tocky Tg4 Tiger (*Il10*-GFP)	Jennings et al., 2020	PMID: 33147449
Mouse: *Nr4a3*-Tocky Great (*Ifng*-YFP) Smart17A	Jennings et al., 2020	PMID: 33147449

(*Continued on next page*)

Continued

REAGENT or RESOURCE	SOURCE	IDENTIFIER
Software and algorithms		
GraphPad Prism 9	GraphPad Inc	https://www.graphpad.com/scientific-software/prism/
FlowJo v10	BD Biosciences	https://www.flowjo.com/solutions/flowjo
R version 4.0	R Core Team	https://www.r-project.org/
Timer angle algorithm	Bending et al., 2018b	PMID: 29941474
Partek Flow	Partek	https://www.partek.com/partek-flow/
BlueBee Software: QuantSeq FWD pipeline	BlueBee	https://lexogen.bluebee.com/quantseq
DESeq2	Love et al., 2014	PMID: 25516281
DOSE	Yu et al., 2015	PMID: 25677125
clusterProfiler	Yu et al., 2012	PMID: 22455463
Ashr	Stephens, 2017	PMID: 27756721
biomaRt	Durinck et al., 2009	PMID: 19617889
VennDiagram	Chen and Boutros, 2011	PMID: 21269502
Other		
Illumina NextSeq 500	Illumina	N/A
BD LSR Fortessa	BD Biosciences	Custom product
BD FACS ARIA III	BD Biosciences	Custom product

RESOURCE AVAILABILITY

Lead contact

Further information and requests for resources and reagents should be directed to and will be fulfilled by the lead contact Dr David Bending (d.a.bending@bham.ac.uk)

Materials availability

The study did not generate new materials. *Nr4a3*-Tocky and Great (*Ifng*-YFP) Smart-17A lines are held under MTA from Dr. Masahiro Ono (Imperial College London; *Nr4a3*-Tocky) and Prof Richard Locksley (UCSF; Great Smart-17A).

Data and code availability

Sequencing data have been deposited at GEO and are publicly available as of the date of publication. Accession numbers are listed in the key resources table. Code and data underlying the major conclusions reported in this paper are available from the lead contact upon reasonable request.

EXPERIMENTAL MODEL AND SUBJECT DETAILS

Mice

Nr4a3-Tocky (Bending et al., 2018b) were mated to Tg4 *Il10*-GFP (Burton et al., 2014) to generate Tg4 *Nr4a3*-Tocky *Il10*-GFP mice as previously described (Jennings et al., 2020). *Nr4a3*-Tocky *Ifng*-YFP (Great Smart-17A) mice (Price et al., 2012) were generated as previously described (Jennings et al., 2020). All animal experiments were approved by the local animal welfare and ethical review body and authorised under the authority of Home Office licenses P18A892E0A and PP3965017 (held by D.B.). Animals were housed in specific pathogen-free conditions. Both male and female mice were used, and littermates of the same sex were randomly assigned to experimental groups.

METHOD DETAILS

In vitro cultures

Single cell suspensions of splenocytes were generated as previously described (Jennings et al., 2021). In Figure S1A splenocytes were activated with 10 μM [4Y] MBP for either 4 or 24 h before analysis of $CD4^+$ T cells for activation markers. For Figure S1B, splenocyte preparations were split in half, with half undergoing naive $CD4^+$ T cells isolation using MoJo magnetic bead negative selection kits (BioLegend), and the other half undergoing $CD90^+$ cell depletion (BioLegend) according to the manufacturer's instructions. Naive T cells were then mixed at a ratio of 1:1 with CD90-depleted splenocytes and stimulated with 1 μM of acetylated [4K] myelin basic peptide Ac-ASQKRPSQR, or [4A] Ac-ASQARPSQR or [4Y] Ac-ASQYRPSQR (custom products from GL Biochem Shanghai) in 10% FBS (v/v) RPMI containing 1% penicillin/streptomycin (Life Technologies) at 37°C and 5% CO_2 for the indicated time points.

Immunisations

Tg4 *Nr4a3*-Tocky *Il10*-GFP mice were immunized through subcutaneous injection of [4Y] MBP peptide (doses stated in figure legends) in a total volume of 200 μL PBS into the flank. For re-challenge experiments, second doses were administered to the contralateral flank in a volume of 200 μL PBS. Mice were then euthanised at the indicated time points, and spleens removed to analyze systemic T cell responses.

Antibody treatments

For *in vivo* blockade experiments, *in vivo* grade anti-PD1 (clone 29F.1A12, BioLegend or Bio X Cell, rat IgG2a), *in vivo* grade anti-Lag3 (clone C9B7W BioLegend, rat IgG1) or hamster anti-CD28 (clone 37.51, kind gift from Prof. Anne Cooke, University of Cambridge) were administered through intraperitoneal injection 30 min before peptide re-challenge. For anti-PD1 and anti-Lag3 experiments an isotype pool control group was used consisting of a 1:1 ratio of rat IgG1 (clone MAC 221, kind gift from Prof Anne Cooke, University of Cambridge) and rat IgG2a (clone MAC 219, kind gift from Prof Anne Cooke, University of Cambridge). For data quality control purposes, successful receptor blockade was confirmed through counterstaining a portion of splenic T cells *ex vivo* with APC or PE-Cy7 conjugated antibodies to PD1 or Lag3 (using the same clone as the blocking antibody). One mouse from the anti-PD1 group was excluded from further analysis in Figure 5I-N due to high amounts of PD1 staining remaining.

Flow cytometry and cell sorting

For analysis of splenic lymphocytes, single cell suspensions were prepared as described above utilizing a red blood cell lysis buffer (ThermoFisher). Cells were washed once and stained in 96-well U-bottom plates (Corning). Analysis was performed on a BD LSR Fortessa X-20 instrument. The blue form of the Timer protein was detected in the blue (450/40 nm) channel excited off the 405 nm laser. The red form of the Timer protein was detected in the mCherry (610/20) channel excited off the 561 nm laser. A fixable eFluor 780-flurescent viability dye (eBioscience) was used for all experiments. The following directly conjugated antibodies were used in these experiments: CD4 Alexa Fluor (AF) 700 (Clone RM4-4, BioLegend), TCRβ Alexa Fluor 700 (clone H57-597, BioLegend), CD4 BUV737 (Clone GK1.5, BD Biosciences) TCR Vβ8.1, 8.2 PerCP-eFluor 710 (Clone KJ16-133, Thermofisher), TCR Vβ8.1, 8.2 BUV395 (clone, MR5-2, BD Biosciences) CD4 BUV395 (Clone GK1.5, BD Biosciences), CD8a BUV395 (clone 53-6.7, BD Biosciences), TCRβ PerCP-Cy5.5 (clone H57-597, Tonbo Biosciences), PD1 APC or PE-Cy7 (clone 29F.1A12, BioLegend), Tigit PE-Cy7 (clone 1G9, BioLegend), Lag3 APC or PE-Cy7 (clone C9B7W, BioLegend), CTLA-4 PE (clone UC10-4B9, BioLegend), OX40 APC (clone OX-86, BioLegend), GITR PE-Cy7 (clone DTA-1, BioLegend), ICOS Alexa Fluor 700 (clone C398.4A, BioLegend), IRF8 PE (clone V3GYWCH, Invitrogen), CD69 APC (clone H1.2F3, BioLegend), CD25 PerCP-Cy5.5 (clone PC61, BioLegend), CD44 AF700 (clone IM7, BioLegend), I-A/I-E PE-Cy7 (clone M5/114/15.2, BioLegend), PD-L1 APC (clone 10F.9G2, BioLegend), rabbit anti-mouse STAT4 (clone 2H9L5, Invitrogen) followed by F(ab')2-Goat anti-Rabbit IgG (H+L) Cross-Adsorbed Secondary Antibody APC (Invitrogen). For intracellular staining of CTLA-4, IRF8 and STAT4, the Foxp3 transcription factor staining buffer kit was used (eBioscience). For cell sorting, single cell suspensions from biological replicate mice were generated and stained individually with distinct CD4 fluorochromes (e.g., AF700, BUV395, BUV737) to permit multiplexing and parallel cell sorting. Cells were sorted on a FACS Aria cell sorter gating on *Nr4a3*-Blue$^+$*Nr4a3*-Red$^-$ for 4-h time point, *Nr4a3*-Blue$^+$*Nr4a3*-Red$^+$ for 12-h time point and *Nr4a3*-Blue$^-$*Nr4a3*-Red$^+$ for the 24-h time point. For cell sorting in Figure 5, cells were sorted for *Nr4a3*-Blue$^+$Red$^+$ T cells. Cells were sorted into 20% FBS RPMI. A small portion of sorted T cells were re-analyzed on the flow cytometer to assess purity. Remaining cells were centrifuged for 5 min at 500 g before 100μL of extraction buffer added (Arcturus Picopure RNA kit, Thermofisher) and lysates frozen at −80°C.

MC38 model

MC38 colorectal cell line (kind gift from Prof. David Withers, University of Birmingham) was passaged in 10% FBS (v/v) RPMI containing 1% penicillin/streptomycin (Life Technologies). On day of experiment, MC38 cells were harvested and resuspended in PBS (Sigma) at a concentration of 2.5 million/ mL and 0.25 million MC38 cells injected sub cutaneously under the right flank of *Nr4a3*-Tocky *Ifng*-YFP (Great) Smart-17A mice in a final volume of 100 μL PBS. Tumor size was measured using callipers. Whole tumors from mice were excised, weighed, and then dissociated using scissors in 1.2 mL of digestion media containing 1 mg/mL collagenase D (Merck Life Sciences) and 0.1 mg/mL DNase I (Merck Life Sciences) in RPMI. Samples were then incubated for 20-25 min at 37°C in a thermoshaker. Digestion mixture was then passed through a 70 μm filter (BD Biosciences) and washed with 30 mL ice cold media (10% FBS RPMI). Suspension was then centrifuged at 1500 rpm for 5 min at 4°C. Pellets were then re-suspended in staining media (2% FBS PBS) for labeling with fluorescently conjugated antibodies.

RNA-seq library preparation and analysis

RNA was extracted from lysates using the Arcturus Picopure RNA kit (ThermoFisher) according to the manufacturer's instructions. 15-25 ng of RNA was used for generation of sequencing libraries using the Quantseq 3' mRNA-seq Library Preparation kit (Lexogen). Briefly, library generation was commenced with oligodT priming containing the Illumina-specific Read 2 linker sequence. After first strand synthesis, RNA was degraded. Second strand synthesis was initiated by random priming and a DNA polymerase. Random primers contained the illumina-specific Read 1 linker sequence. Double stranded DNA was purified from the reaction using magnetic beads and libraries amplified and sequences required for cluster generation and sample indexes were introduced. Libraries were normalized and pooled at a concentration of 4 nM for sequencing. Libraries were

sequenced using the NextSeq 500 using a Mid 150v2.5 flow cell. Cluster generation and sequencing was then performed and FASTQ files generated. FASTQ files were then downloaded from the Illumina base space and uploaded to the BlueBee cloud for further analysis (Lexogen). FASTQ files were merged from the 4 lanes to generate final FASTQ files which were loaded into the BlueBee QuantSeq FWD pipeline. FASTQC files were generated and Bbduk v35.92 from the bbmap suite was used for trimming of low-quality tails, poly(A)read-through and adaptor contamination. STAR v2.5.2a aligner was used for alignment of reads to the mouse GRCm38 (mm10) genome. HTSeq-count v0.6.0 was used to generate read counts for mRNA species and mapping statistics. Raw read counts in the .txt format were used for further analysis using DESeq2 (Love et al., 2014) in R version 4.0. DESeq2 estimates variance-mean dependence in count data from high-throughput sequencing assays and tests for differential expression based on a model using the negative binomial distribution. A DESeq dataset was created from a matrix of raw read count data. Data were filtered to remove genes with fewer than 10 reads across all samples. Log2 fold change estimates were generated using the DESeq algorithm and shrinkage using normal (Figure 2) or the ashr algorithm (Figure 5) (Stephens, 2017) to estimate log2 fold changes (lfc). Principal component analysis identified one replicate batch in the effects of checkpoint blockade (Figure 5) to be an outlier and these three samples (which had been sorted and processed as a batch) were not included in further analysis. Differentially expressed genes (DEGs) were selected based on an adjusted p value of < 0.05, and a lfc greater > 1 for Figure 2, or any gene with an adjusted p value of < 0.05 for Figure 5. Normalized read counts were transformed using the regularised log (rlog) transformation. This function transforms the count data to the log2 scale in a way which minimizes differences between samples for rows with small counts, and which normalizes with respect to library size. Heatmap analysis was performed on the rlog transformed data using the R package gplots. For KEGG pathway analysis clusterProfiler (Yu et al., 2012), DOSE (Yu et al., 2015), and biomaRt (Durinck et al., 2009) packages were used.

Analysis of published human anti-PD1 and MC38 anti-PD-L1 datasets

Genes upregulated in the Riaz cohort of human melanoma patients receiving nivolumab compared to pre-therapy samples were stratified into two groups as in GEO: GSE 91061 (Riaz et al., 2017). On therapy group (**OT**, n = 76) consisted of genes upregulated compared to pre-therapy in patients regardless of clinical responses. Responder genes (**Res**, n = 501) were those upregulated in patients showing clinical response to treatment compared to pre-therapy. For this analysis a lfc > 0.5 and adjusted p value < 0.1 was set. The R package VennDiagram (Chen and Boutros, 2011) was used for analysis of overlapping genes between the genes in the **OT**, **Res** and genes identified in this study upregulated at 4 h in 80 μg versus 0.8 μg immunized mice (**4 h**, n = 337) or genes upregulated 4 h after re-challenge in the presence of anti-PD1 *in vivo* (**PD1**, n = 51). For analysis of the validation cohort from (Gide et al., 2019), raw FASTQ files for patients early during treatment (EDT) were downloaded from ENA: PRJEB23709 and analyzed using Partek Flow software (Partek). Briefly, raw reads were trimmed then aligned to the hg38 genome using Star Aligner. Partek Annotation E/M model was used to generate gene counts using the reference ensemble release 99 with automatic detection of strandedness. Gene level FPKM values were then extracted.

For analysis of gene expression in response to 0.5 mg anti-PD-L1 (clone 10F.9G2) therapy compared to IgG2b control in mice inoculated with 0.5 million MC38 cells, raw count expression data was kindly provided by Dr Mirjana Efremova (Efremova et al., 2018) from GEO: GSE93018. Differentially expressed genes (DEG) were identified using DESeq2 as described above. For this analysis genes were considered DEG with a fold change > 1.5 and an adjusted p value < 0.05. Normalized read counts were transformed using the regularised log (rlog) transformation. Heatmap analysis was performed on the rlog transformed data using the R package gplots.

Generation and implementation of TCR.strong metric

FPKM for *TNFRSF4*, *ICOS, IRF8*, *TNIP3* and *STAT4* were extracted for melanoma patients from supplementary files appended to GEO: GSE 91061 (Riaz et al., 2017), or generated as described earlier from (Gide et al., 2019). FPKM were converted to TPM as described (Pachter, 2011) through dividing each gene level FPKM by the sum of all FPKM in annotated genes within that sample. This figure was then multiplied by 1^e6 then offset by 0.01 to avoid 0 values. For analysis of MC38 model responses, the gene level counts per million (CPM) was utilized with a 0.01 offset to calculate TCR.strong and T activation scores. The geometric means of the TPM for *TNFRSF4, ICOS, IRF8, TNIP3* and *STAT4* (TCR.strong) or *NR4A1, CD69, CD25, TNFRSF9* (T activation) was then calculated for each patient (Table S5 and S6). Patient responses were characterized as: complete remission (CR), partial remission (PR), stable disease (SD) or progressive disease (PD) as per (Riaz et al., 2017) and (Gide et al., 2019). Responder groups were classified as patients displaying CR, PR, and SD for analyses. Non-responders were classified as PD patients. Clinical outcome data for Riaz et al. cohort was extracted from supplemental data from (Riaz et al., 2017) and the github repository https://github.com/riazn/bms038_analysis/tree/master/data. Patients with missing disease outcomes or non-evaluated (NE) statuses were excluded from analysis in Figure 7. Clinical outcomes for patients in the Gide cohort were extracted from supplemental data tables from (Gide et al., 2019).

QUANTIFICATION AND STATISTICAL ANALYSIS

Sequencing data analysis is described earlier. For non-sequencing data analysis, statistical analysis was performed on Prism 9 (GraphPad) software. For comparison of more than two means over time, a two-way ANOVA with Tukey's or Sidak's multiple comparison's test was used. For comparison of Kaplan Meier survival curves, the TCR.strong scores or T activation scores for the group of patients on therapy with reported survival data were split at the median value into "High" and "Low" scores, and data analyzed

using a Log-rank (Mantel-Cox) test. For a comparison of more than two means, a one-way ANOVA with Tukey's multiple comparisons test was used. For comparison of non-parametric data, a Mann Whitney U test was performed. Variance is reported as mean ± SEM unless otherwise stated; data points typically represent individual mice or patients. Normalized *Nr4a3*-Timer Blue, *Nr4a3*-Timer Red, active TCR signaling and mean Timer angles were generated as previously described using custom algorithms (Bending et al., 2018b). Flow cytometry data were analyzed using FlowJo software (BD Biosciences). *p = < 0.05, **p = < 0.01, ***p = < 0.001, ****p = < 0.0001.

Immunity, Volume *54*

Supplemental information

Antigen and checkpoint receptor engagement recalibrates T cell receptor signal strength

Thomas A.E. Elliot, Emma K. Jennings, David A.J. Lecky, Natasha Thawait, Adriana Flores-Langarica, Alastair Copland, Kendle M. Maslowski, David C. Wraith, and David Bending

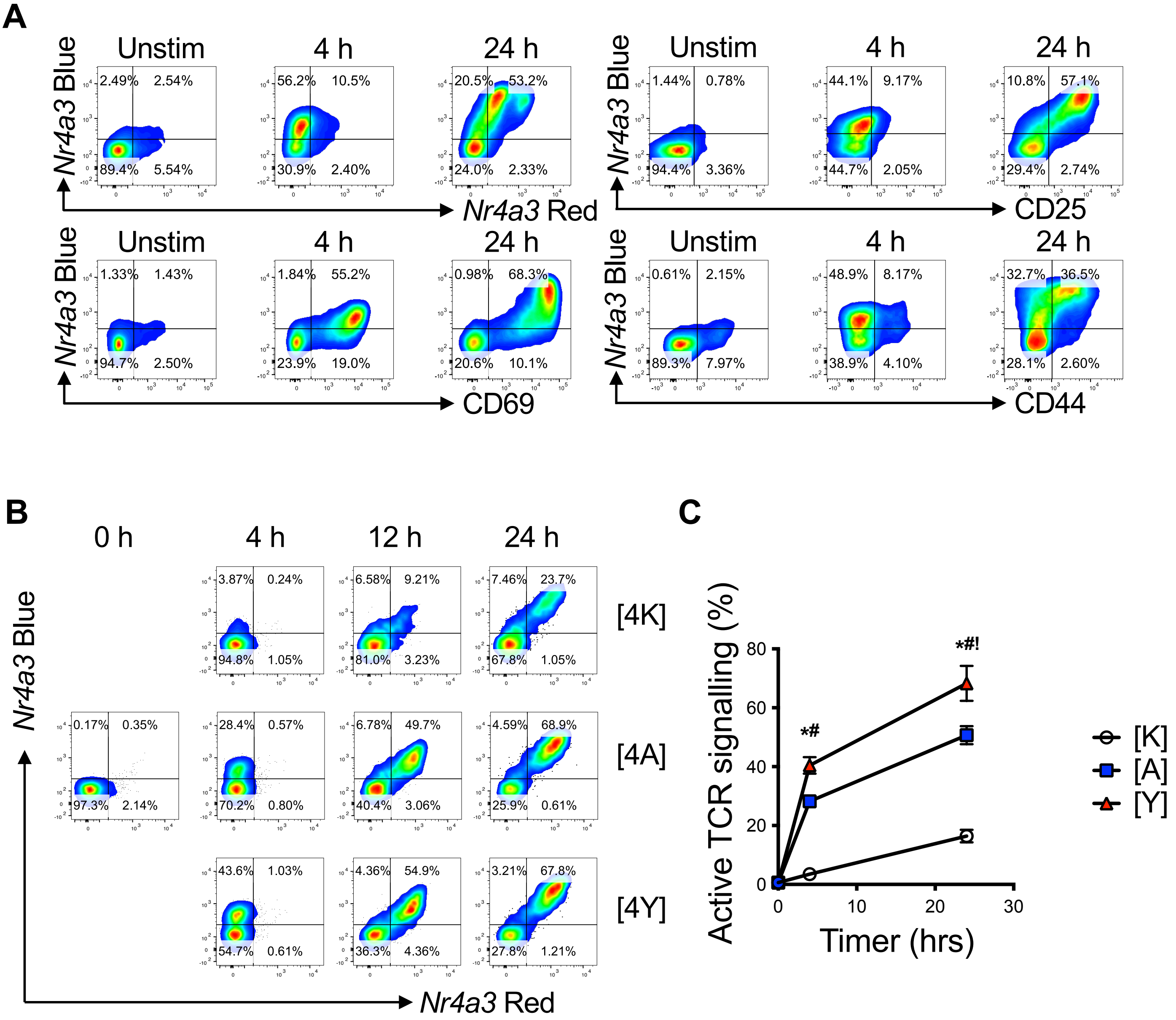

Supplementary Figure 1: Modified MBP peptide variants induce potent T cell activation in vitro (related to Figure 1)

(**A**) Splenocytes from Tg4 *Nr4a3-Tocky Il10*-GFP mice were incubated with 10 µM of [4Y] MBP peptide for the indicated times before analysis of CD4⁺ Tg4 T cells by flow cytometry for the markers shown. (**B**) Naïve CD4⁺ T cells from Tg4 *Nr4a3*-Tocky *Il10*-GFP mice were incubated with CD90-depleted splenocytes in the presence of 1 µM of native [4K] MBP peptide, or [4A] or [4Y] variants for the times indicated before analysis of *Nr4a3*-Blue vs *Nr4a3*-Red expression in CD4⁺ Tg4 T cells. (**C**) Summary data showing the % *Nr4a3*-Blue⁺ in CD4⁺ T cells in the 3 peptide groups. MBP [4K] = white circles, MBP [4A] = black squares and MBP [4Y] = red circles. Bars represent mean ±SEM, n=3. Statistical analysis by two-way ANOVA with Tukey's multiple comparisons test. Significant differences between [4Y] and [4K] = *, [4A] and [4K] = #, or [4Y] and [4A] = !.

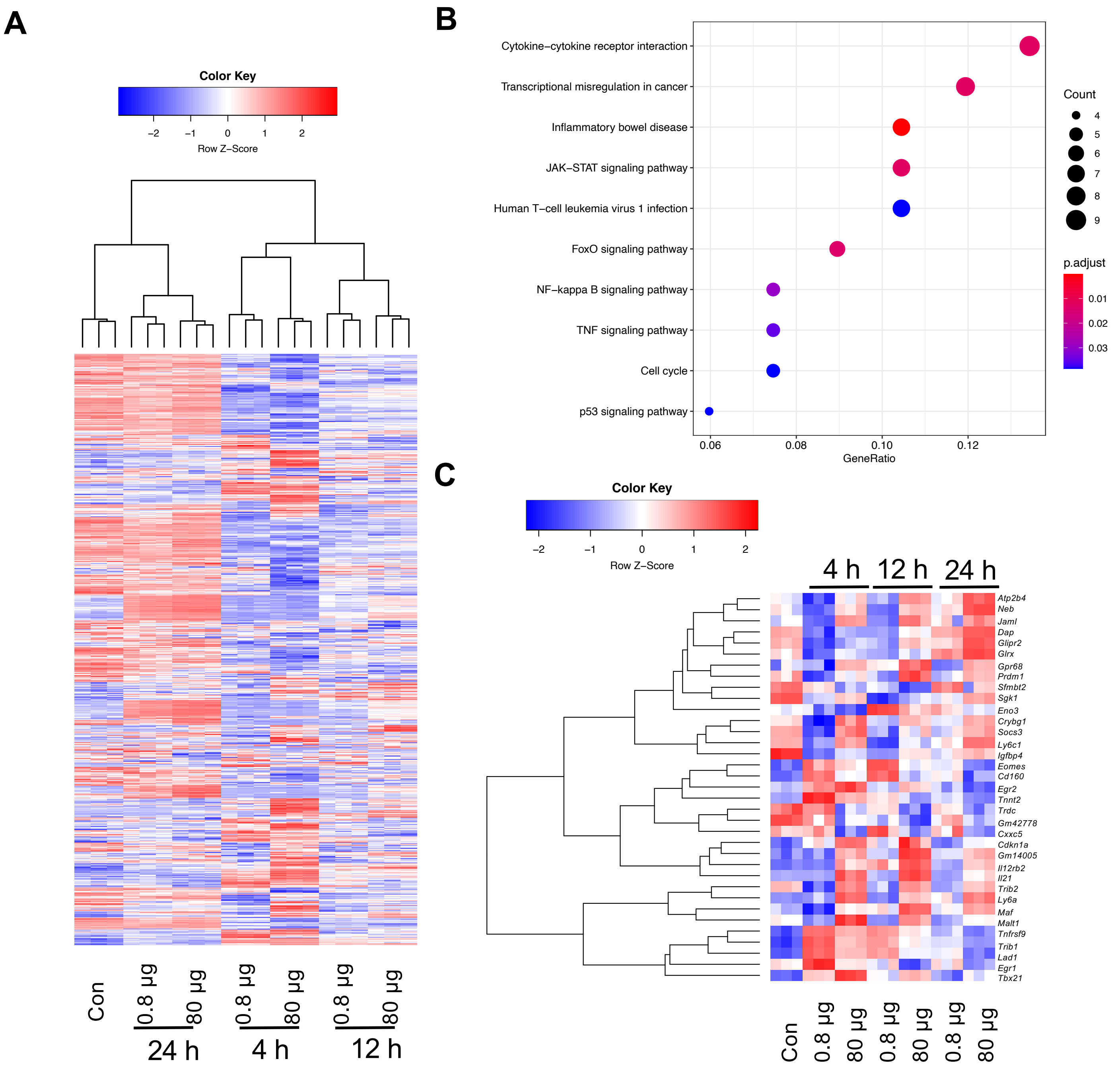

Supplementary Figure 2: Analysis of DEGs in T cells receiving strong or weak TCR signalling in vivo (related to Figure 2)

(A) Z-score heatmap analysis of log2 transformed and normalised counts for all unique DEG identified between 0.8 and 80 µg groups at 4, 12 or 24 h. (**B**) KEGG pathway analysis of DEG at 24 h between 0.8 µg and 80 µg [4Y] MBP immunised mice. (**C**) Z-score heatmap analysis of log2 transformed and normalised counts for genes that show differential expression across all 3 time points (4, 12 and 24 h) analysed between 0.8 µg and 80 µg immunised groups.

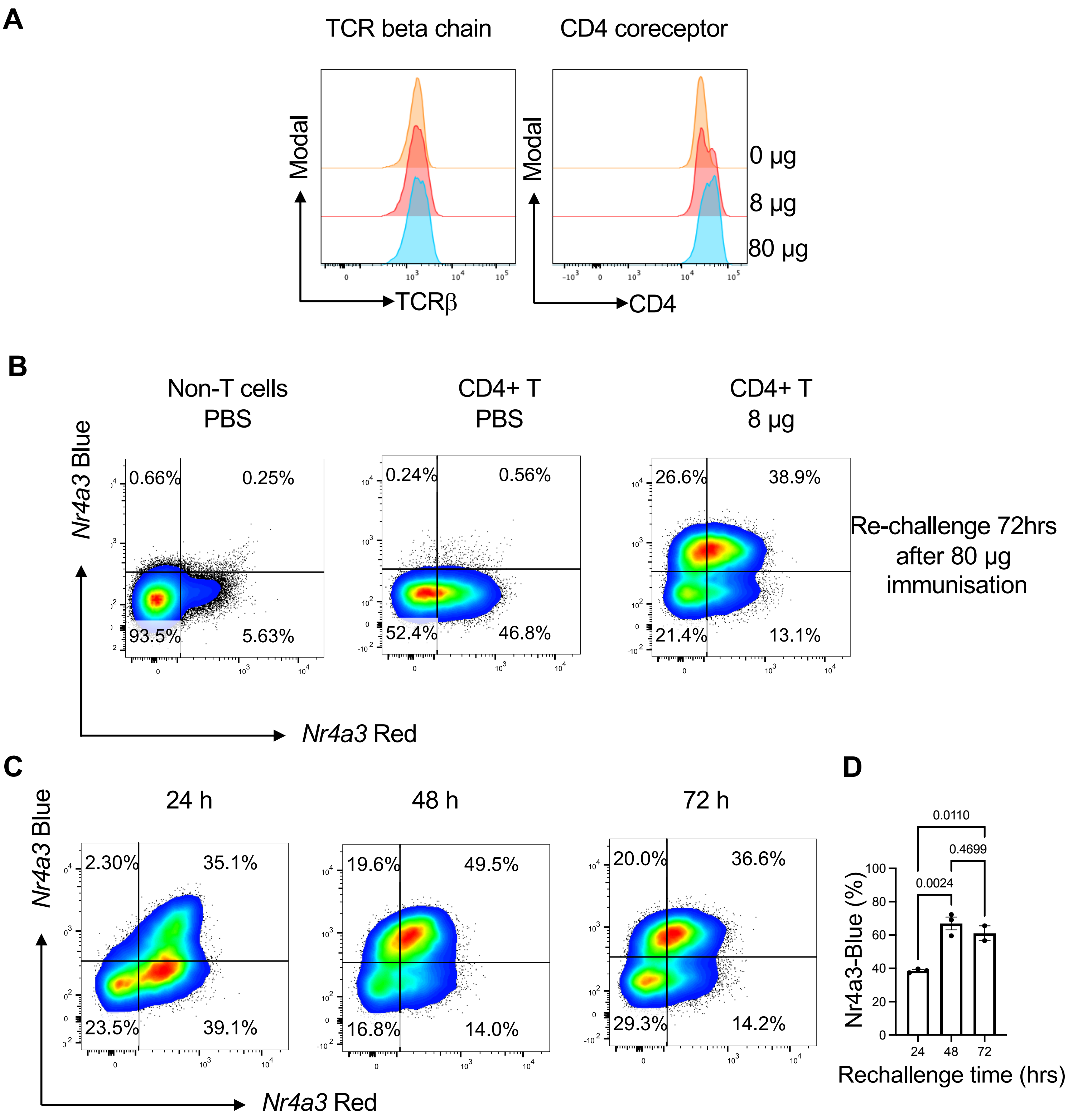

Supplementary Figure 3: Reduced *Nr4a3* activation is not related to TCR levels but is affected by the time between stimulations (related to Figure 4)
(A) Tg4 *Nr4a3*-Tocky *Il10*-GFP mice were immunised s.c. with 0 µg, 8 µg or 80 µg of [4Y] MBP peptide for 24 h. Mice were then rechallenged with 8 µg [4Y] MBP for four h before splenic CD4⁺ *Nr4a3*-Timer⁺ T cells were analysed for their expression of TCRβ and CD4. (**B**) Tg4 *Nr4a3*-Tocky *Il10*-GFP mice were immunised s.c. with 80 µg [4Y] MBP. 72 h later mice were rechallenged with 8 µg or PBS and then splenic CD4⁺ T cell responses analysed for *Nr4a3*-Blue vs *Nr4a3*-Red expression. (**C**) Tg4 *Nr4a3*-Tocky *Il10*-GFP mice were immunised s.c. with 80 µg of [4Y] MBP. Mice were then rechallenged at the indicated time points with 8 µg [4Y] MBP and analysed 4 h later for *Nr4a3*-Blue vs *Nr4a3*-Red expression. (D) Summary data from (C). Bars represent mean ± SEM, statistical analysis by one-way Anova, with Tukey's multiple comparisons test.

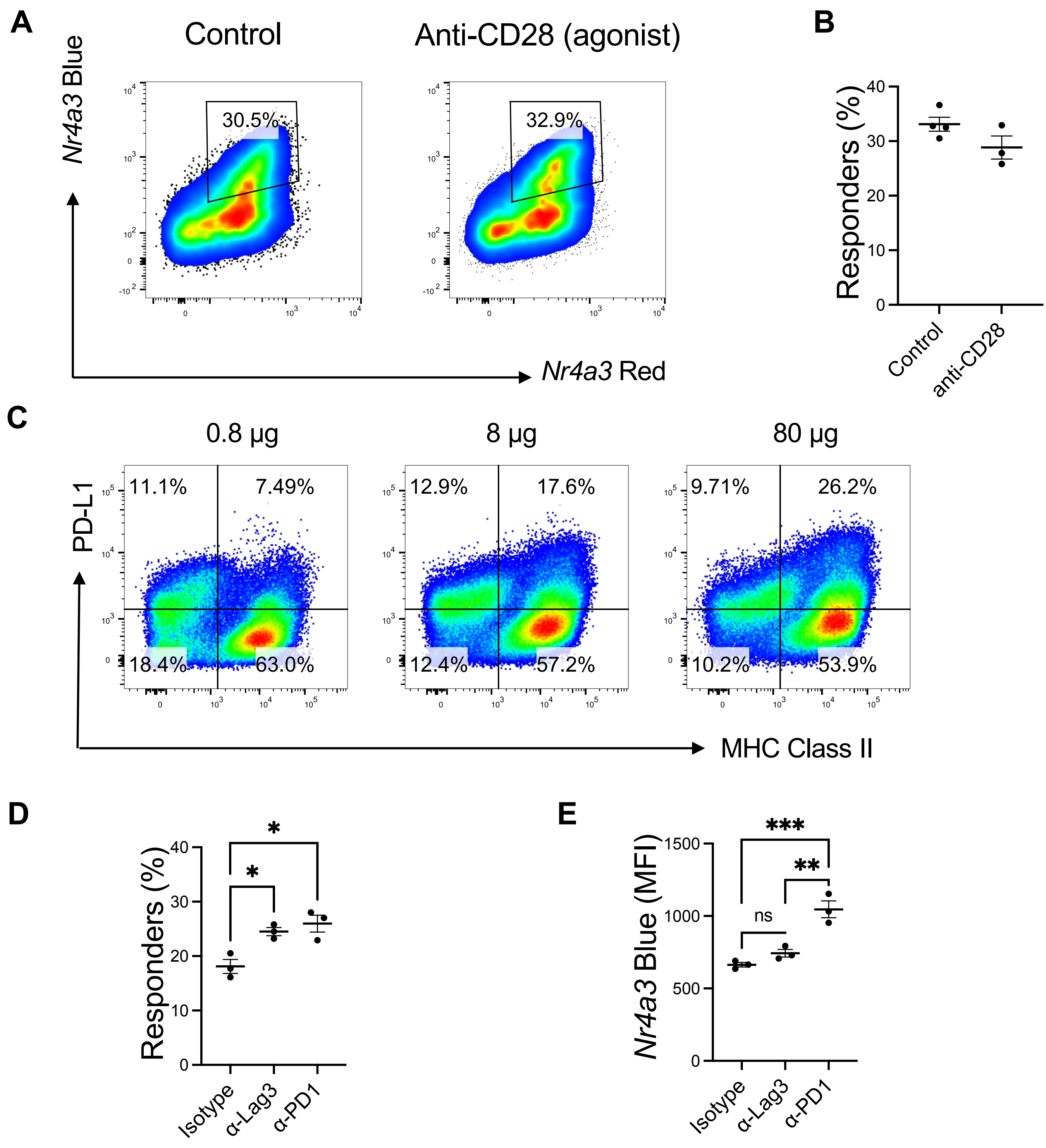

Supplementary Figure 4: Effects of CD28, Lag3 and PD1 pathways on T cell re-activation in vivo (related to Figure 5)

(**A**) Tg4 *Nr4a3*-Tocky *Il10*-GFP mice were immunised s.c. with 80 µg of [4Y] MBP. 24 h later mice were randomised to receive either PBS or 0.5 mg agonistic anti-CD28 30 minutes prior to re-challenge with 8 µg [4Y] MBP peptide. Splenic CD4+ T cells were analysed for *Nr4a3*-Blue vs. *Nr4a3*-Red analysis 4 h after peptide rechallenge. (**B**) Summary data of (**A**), control n=4, anti-CD28 n=3. (**C**) Tg4 *Nr4a3*-Tocky *Il10*-GFP mice were immunised s.c. with 0 µg, 8 µg or 80 µg for 24 h before analysis of live splenocytes for MHC Class II versus PD-L1 expression. (**D&E**) Tg4 *Nr4a3*-Tocky *Il10*-GFP mice were immunised s.c. with 80 µg of [4Y] MBP. 24 h later mice were randomised to receive either isotype, anti-Lag3 or anti-PD1 30 minutes prior to re-challenge with 8 µg [4Y] MBP peptide. The frequency of responder (*Nr4a3*-Blue^{+}Red^{+}) T cells (**D**) or *Nr4a3*-Blue Median expression in responder (*Nr4a3*-Blue^{+}Red^{+}) T cells (**E**) 4 h after peptide rechallenge are shown. N=3, dots represent individual mice, bars represent mean ±SEM. Statistical analysis by one-way ANOVA with Tukey's multiple comparisons test. *=p<0.05, **=p<0.01, ***=p<0.001.

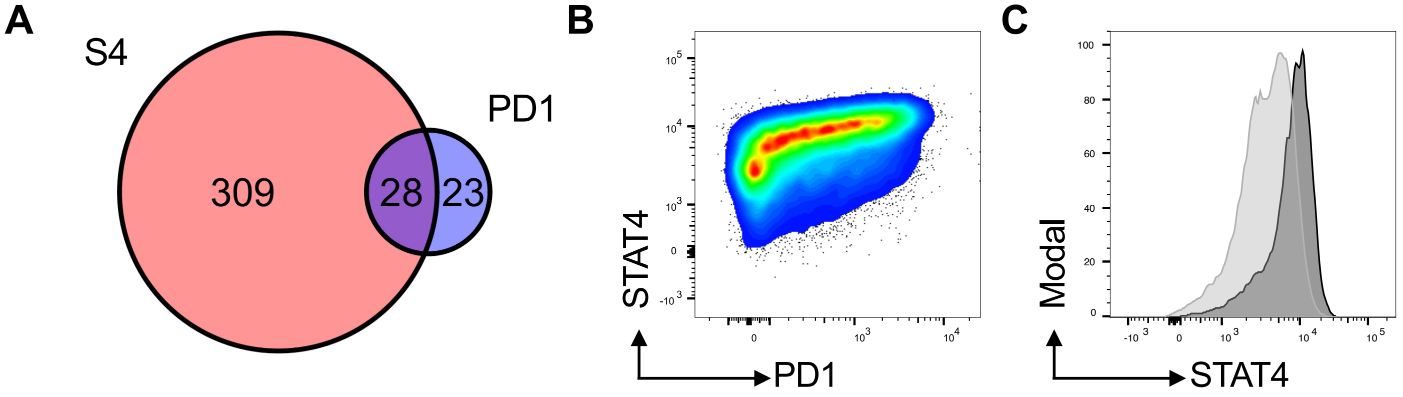

Supplementary Figure 5: Majority of anti-PD1 specific T cell genes are upregulated in T cells receiving strong TCR signal, and STAT4 is increased in activated T cells (related to Figures 2, 5 and 6)

(**A**) Genes upregulated in Tg4 CD4$^+$ T cells 4 h after receiving 80 µg vs 0.8 µg [4Y] MBP (Figure 2) were intersected with genes selectively upregulated at 4hrs of T cells re-activated in the presence of anti-PD1 in vivo (Figure 4). 28 out of 51 genes were overlapping and utilised to interrogate MC38 tumour responses in Figure 6. (**B**) Tg4 *Nr4a3*-Tocky *Il10*-GFP mice were immunised s.c. with 80 µg of [4Y] MBP. 24 h later mice received 0.5 mg rat IgG2a 30 minutes prior to re-challenge with 8 µg [4Y] MBP peptide. 4 h later intracellular STAT4 and PD1 expression was analysed in splenic CD4$^+$ T cells. (**C**) STAT4 levels in PD1^{hi} (black histogram) or PD1^{lo} (grey histogram) from CD4$^+$ T cells in (**B**).

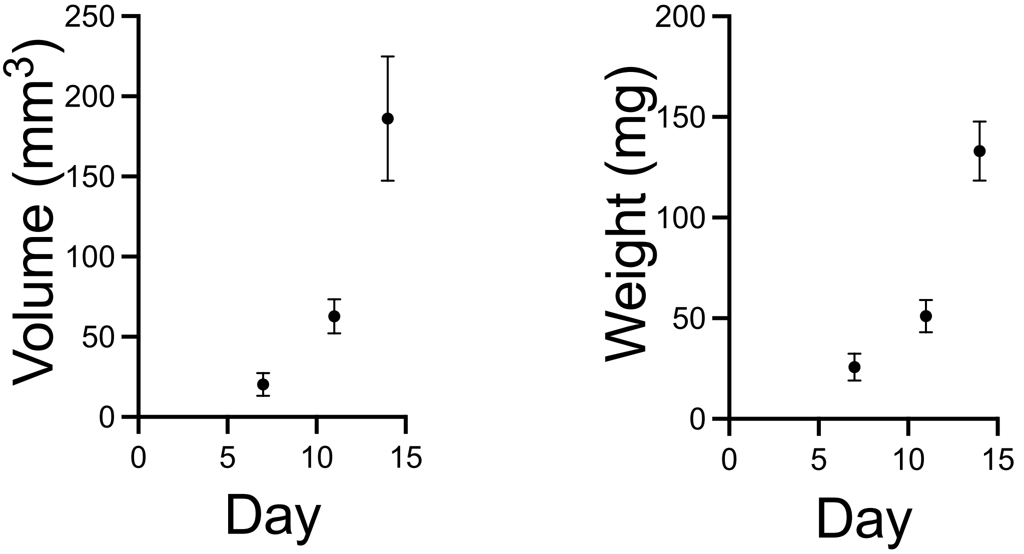

Supplementary Figure 6: MC38 tumour volume and weight (related to Figure 6)

Nr4a3-Tocky *Ifng*-YFP mice were injected with 0.25 M MC38 cells and tumours measured with callipers at the indicated times or weighed upon sacrificing of the mice. N=3, bars represent mean ±SEM.

A

TCR.strong genes
TNFRSF4, ICOS, IRF8, TNIP3, STAT4

T activation genes
CD69, IL2RA, NR4A1, TNFRSF9

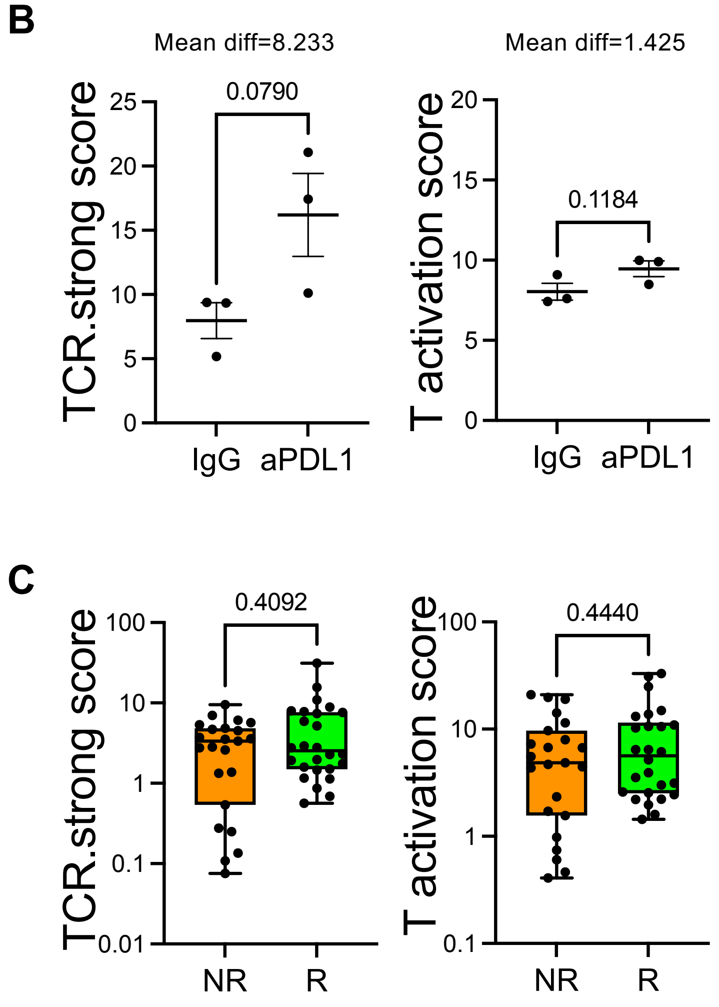

Supplementary Figure 7: TCR.strong and T activation metric analysis (related to Figure 7)

(A) Genes included in the TCR.strong and T activation gene metrics. (**B**) TCR.strong and T activation scores for mice in Figure 6 E&F from (Efremova et al., 2018). Statistical analysis by student t test. (C) Analysis of pre-therapy TCR.strong (left) or T activation (right) scores in patients from the Riaz et al. cohort divided into non responder (NR, orange) and responder (R, green). Dots represent individual patients. Statistical analysis by Mann Whitney U test.
